# Outcomes of orangutan wild-to-wild translocations reveal conservation and welfare risks

**DOI:** 10.1371/journal.pone.0317862

**Published:** 2025-03-19

**Authors:** Julie Sherman, Maria Voigt, Marc Ancrenaz, Erik Meijaard, Felicity Oram, Elizabeth A. Williamson, Anne E. Russon, David J. I. Seaman, Christine Caurant, Dirck Byler, Serge A. Wich

**Affiliations:** 1 Wildlife Impact, Portland, Oregon, United States of America; 2 HUTAN, Sandakan, Sabah, Malaysia; 3 Borneo Futures, Bandar Seri Begawan, Brunei Darussalam; 4 OrangJUGA, Kota Kinabalu, Sabah, Malaysia; 5 Faculty of Natural Sciences, University of Stirling, Stirling, United Kingdom; 6 Department of Psychology, Glendon College, York University, Toronto, Canada; 7 Durrell Institute of Conservation and Ecology, School of Anthropology and Conservation, University of Kent, Canterbury, United Kingdom; 8 Independent Conservation Consultant, Lannion, France; 9 IUCN SSC Primate Specialist Group Section on Great Apes, c/o Re:wild, Austin, Texas, United States of America; 10 Re:wild, Austin, Texas, United States of America; 11 School of Biological and Environmental Sciences, Liverpool John Moores University, Liverpool, United Kingdom; Cholistan University of Veterinary and Animal Sciences, PAKISTAN

## Abstract

Wild orangutans (*Pongo* spp.) are captured and moved (wild-to-wild translocated) primarily to prevent crop foraging or out of concern for orangutans’ survival in fragmented habitat. Little is known about wild-to-wild translocation frequency, circumstances, and possible species conservation and individual welfare outcomes. We investigated orangutan wild-to-wild translocations in Indonesia from 2005 to 2022 using primarily data from public sources and consultation with practitioners. At least 988 wild orangutans were captured for translocation during the study period, including many reproductively valuable resident females and adult males removed from unprotected fragmented forests and forest patches. Data on health condition (n = 808) indicated 81.7% were reported as healthy at time of capture. Information on post-capture disposition (n = 268) showed that only 23% were translocated immediately. Mean estimated killing combined with reported translocation removals was calculated to affect 3.3% of orangutans in Kalimantan, and 11.6% in Sumatra, both higher than the threshold of mortality from human actions expected to drive populations to extinction. Negative impacts are likely compounded where multiple individuals are translocated from the same area, and for the Tapanuli orangutan (*P. tapanuliensis*), which has the smallest population and range of all orangutan species. Data on reasons for capture (n = 743) indicated most translocations (69%) were conducted to address crop foraging and orangutan presence in or around croplands and plantations. Forest cover analysis around 104 orangutan capture sites with high resolution spatial information indicated that deforestation levels in the year preceding capture were not significantly associated with likelihood of captures for translocation. To improve conservation outcomes, wild-to-wild translocations should be used only in exceptional circumstances. Most orangutans should instead be monitored and protected in situ by addressing conflicts and maintaining the forests, including forest fragments, they are using. When translocation is necessary, post-release survival and potential conservation impacts must be monitored.

## Introduction

Conservation translocations are human-mediated transfers of organisms from one site to another for the purpose of protecting species and ecosystems [e.g., 1]. Wild-to-wild translocation entails capturing individual wild animals from areas where they are found and moving them to other sites within their species’ range. Wild-to-wild translocation has become a common strategy to address or pre-empt negative interactions between humans and wildlife, and to remove wildlife from areas where humans prefer it not to be [e.g., 2–7]. While wild-to-wild translocations may remove individuals from situations where their lives are in immediate danger from humans, they have limited long-term success in mitigating human-wildlife conflicts and, in many cases, also have negative impacts on post-release survival and welfare of released individuals and on species conservation [8]. Unintended outcomes of wild-wild translocations undertaken to ameliorate conflicts include injury or death of individual animals during capture, translocation or release, and released individuals being killed by conspecifics or humans [e.g., 7,9,10]. Released individuals often return to sites from which they were removed, or cause conflict with humans in new areas [e.g., 2,6,11]. Furthermore, translocations have resulted in inadvertent genetic mixing of species and subspecies, mixing of traits and local cultures specific to source populations, as well as disease transmission and negative social impacts, including competition with resident conspecifics [e.g., 12–16].

Orangutans (*Pongo* spp.) are severely threatened by conversion and fragmentation of their habitats to agricultural plantations, forestry, mining, hydropower, and other infrastructure, and by illegal killing, capture and trade [17–19]. For over three decades, the three species of orangutans, *Pongo pygmaeus* (Bornean orangutan), *Pongo abelii* (Sumatran orangutan), and *Pongo tapanuliensis* (Tapanuli orangutan), all listed as Critically Endangered by the International Union for the Conservation of Nature (IUCN) Red List of Threatened Species [20–22], have been wild-to-wild translocated in large numbers. Orangutans are translocated for the following reasons: 1) they are deemed at risk from human attacks or forest fires; 2) they are found in habitats perceived as unsuitable, such as small or isolated forest fragments in agricultural and other anthropogenic landscapes; 3) they are feeding on or otherwise damaging cultivated crops, or are assumed likely to do so; 4) they are deemed a risk to human safety or property; 5) they are in the path of planned forest clearing or development or considered at risk from potential deforestation; or 6) they are believed to be suffering from malnutrition [5,11]. The distances orangutans are moved during translocations vary considerably, from a few kilometers to hundreds of kilometers from their capture sites [5, this study]. Limited empirical evidence is available to evaluate wild-to-wild translocation impacts on orangutans. A compendium of primate conservation actions [23] found a single published study related to orangutan wild-to-wild translocation, which reported translocation of one population of 84 animals, without post-release survival data [24]. Orangutans are semi-solitary foragers but live in diffuse communities of related or familiar individuals, with philopatric resident females and dispersing males [16,25]. Female orangutans are strongly tied to their natal areas, generally intolerant of unrelated females and may be aggressive towards them [26], and may pose a potential threat to the infants of unrelated females [27]. Males compete intensively for mating access, while females mate with multiple males, flanged and unflanged, for each conception [28]. Immature orangutans are wholly dependent on their mothers from birth until they are around seven to eight years old, a period when they learn how to process food and where and when to find it, after which they stay within their matrilineal home range refining these skills until they reach around 15 years of age [29,30].

Free-ranging orangutans live in intact tropical moist forests, degraded and selectively logged forests and mosaic habitats (mixed forest types or forest mixed with other vegetation types), and will use agricultural plantations, farms, gardens, and exotic tree plantations [31–36]. Females have been observed occupying small forest fragments for decades and are regularly seen with dependent infants and semi-independent juvenile offspring [37]. Orangutans can maintain viable population densities in mosaic landscapes that include forest, industrial tree plantations, production forests, and diversified agricultural areas [19,38–40]. Human food crops may be easier for orangutans to process and may have higher sugar and nutritional content, making them very attractive to orangutans [see: 41], especially ranging males, even if sufficient natural foods are available in their home range [42]. Orangutans will also consume oil palm shoots and fruits [35]. More than 75% of orangutans are estimated to live outside protected areas in Sumatra and Indonesian Borneo [43,44]. Although generally an arboreal species, orangutans travel significant distances on the ground, even in their natural habitats [45,46].

Threats to orangutans and their habitats, notably land-cover changes and conflicts with humans, are taking place across the species’ ranges [19,47,48]. People increasingly report that orangutans are in or near farms, gardens, plantations, infrastructure developments, and other human-use areas [49,50]. Removing these individuals by translocating or killing them increases population fragmentation and jeopardizes viability of the metapopulation [37,44,51]. A recent modeling analysis by Seaman et al. [51] found that removing even very small numbers of orangutans through translocation or killing had marked negative effects on population viability. Rates of orangutan offtake by killing or capture and removal from translocation totaling 2% of the species population were projected to lead to long-term population declines averaging 76% over 250 years, while rates of 4% or higher were projected to cause functional extinction [51]. Similarly, an orangutan population viability analysis found that human-caused mortality affecting 1% of the species population would drive population extirpation in sub-optimal habitats, with even slightly higher mortality driving extirpation in higher quality habitats [52]. To better understand the effects of capturing and removing individuals from wild orangutan populations, we examined orangutan wild-to-wild translocations in light of current understanding of their socioecology, as well as threats and conservation management. Although our data on orangutan translocations are incomplete, public attention to the species and activities affecting its conservation have resulted in a large body of information compared to that available for wild-to-wild translocations of many other taxa. This case study thus adds to the body of knowledge on the uses and outcomes of wild-to-wild translocation and presents methods that can be adapted to assess translocation impacts for a broad range of taxa.

To assess the effects and better evaluate the necessity of translocations, we collected data on and reviewed circumstances of wild-to-wild translocations of the three Critically Endangered species of orangutans (*Pongo* spp.) in Indonesia. We investigated the following questions: 1) what were the age, sex and physical condition of the individuals captured, 2) were captured individuals translocated, and if so, where to, 3) what habitat types were orangutans encountered and captured in, and what were the conditions that led to the perceived need for translocation, 4) was deforestation related to perceived need for translocation and did deforestation change following translocations, and 5) what were the outcomes for species conservation and welfare. This study investigates orangutan wild-to-wild translocation of all three species using primarily public data combined with input from practitioners and a replicable mapping approach to consider landscape changes. We provide decision-making recommendations to prevent unnecessary and potentially harmful wild-to-wild translocations. Our findings point to the urgent need to better monitor, evaluate and manage wild-to-wild translocations to mitigate negative impacts to species and habitats. The process and recommendations herein can be applied to many other species that co-exist with humans.

## Methods

We collected data on orangutans captured for wild-to-wild translocation between 2005 and 2022 in Kalimantan and Sumatra, Indonesia, from published sources (including scientific articles, newspaper articles, annual reports, grant reports, news blogs, charity commission reports, and conference presentations), unpublished sources, and direct communications with translocation practitioners and orangutan researchers (S1 Table). We communicated with 54 practitioners and asked about their experiences with wild-to-wild translocations, the circumstances in which they captured and translocated orangutans, the factors that went into these decisions, and their views on expectations of the local communities and land managers they worked with. Sources of information on specific instances are generally not cited here to maintain confidentiality. Orangutan translocations in Sabah and Sarawak, Malaysia, are not included due to lack of available data.

We used the metric of ‘wild orangutans captured for translocation’ because not all captures resulted in translocation and, although rare, some wild individuals were captured expressly to provide medical care. After captures for translocation, some animals were not released because they died or were not suitable for release due to physical impairment, disease, or lack of appropriate release sites. In other cases, whether and where individuals were released was unreported or unclear. Our dataset underestimates the total number of orangutans captured from the wild for translocation because it includes only captures that could be confirmed as unique instances, so that each capture was counted only once. Many wild-to-wild translocations are not publicly reported [5] because this action is considered sensitive by either practitioners or government entities. For example, we found no published records of wild-to-wild translocations in any province of Sumatra between 2005 and 2011, although given subsequent translocation rates, it is highly unlikely that no orangutans were translocated during these years.

For each capture instance, we collated all available information on the number of orangutans, their age, sex, and physical condition, the location where they were captured, reasons for capture, information about the individual orangutan’s history plus human activities in the area, whether they were translocated and when, and the release location. Capture records usually highlighted any injuries or illness, thus we assumed animals were healthy unless otherwise noted. Trends in the annual numbers of orangutans captured for translocation were assessed using the Mann-Kendall trend test [53], with serial autocorrelation in annual numbers checked using the Ljung box test [54]. Both tests were run in R [55].

We calculated estimated numbers of individuals removed from wild orangutan populations through captures for translocation. We compiled available data on population changes disaggregated by species, geographic and administrative boundaries. Mean annual rates of orangutan population change were calculated by averaging the annual declines shown by survey estimates and modeling projections in Santika et al. [38], Voigt et al. [19], Wich et al. [18], and Utami-Atmoko et al. [43] (S2 Table). Although lack of precise location data for most captures prevents analysis of impacts to most local populations, localized effects were explored through analysis of captures within village administrative boundaries (*desa*) across the orangutan distribution. Village location data were available for 53% of the captures reported (n = 528 of 988). We also analyzed captures of Tapanuli orangutans to look at species-level effects. Standard deviations and confidence intervals for mean population estimates and annual translocation rates were generated in R [55]. We estimated mortality rates from killing of each orangutan species using data on killings detected from 2007 to 2019 at likely wildlife crime detection rates of 1.2–10% per Sherman et al. [56].

Global navigation satellite system (GNSS) coordinates are usually collected at locations where orangutans are captured for translocation, and at the locations they are translocated to, but these coordinates are not always reported publicly. Coordinates were available for 104 (10.5%) of capture records during the study period. Notably, the GNSS coordinates may not be indicative of the precise location at which an orangutan was foraging or living. The individual may have been encountered while it was traveling or been driven away from its original location by human actions, or the recorded point may have been a meeting point or key landmark to enable authorities and translocation practitioners to find the location (F. Oram, unpublished data). Translocation release site coordinates were available for only 47 captured orangutans (4.8% of capture records).

We mapped the 104 captures with GNSS coordinates to investigate how multiple captures might affect local populations and metapopulations. We assessed the habitat context of captures by quantifying forest cover changes between 1999 and 2020 around the recorded capture sites. We used the tropical moist forest, degraded forest, and forest regrowth classes from the annual change collection from Vancutsem et al. [57] at https://forobs.jrc.ec.europa.eu/TMF/explorer, and extracted them in a 5-km circular zone around the capture coordinates. This 5-km buffer is based on the minimum distances orangutans are known to travel through plantations and other human-altered landscapes [M. Ancrenaz, unpublished data; 35,44,51]. We extracted annual forest cover and loss within the buffer and used a Generalized Linear Model (GLM) framework to examine the influence of forest loss on the likelihood of capture events in subsequent years in and around sites where orangutans were captured. Given the high correlation (correlation coefficient > 0.7) between the percentage of intact forest loss one to four years prior, we limited our analysis to evaluating the influence of forest losses that occurred one and five years before the capture events. For captures between 2005 and 2017, we also looked at forest cover changes within five years after capture.

We exemplified translocation circumstances and habitat changes in the 5-km buffer for nine orangutan capture sites (five in Sumatra, and four in Borneo). These nine sites were selected based on availability of capture coordinate data and details on capture circumstances for each of the three orangutan species. For distance from orangutan capture location to release location, we used R [55] and sf package [58,59] to measure the shortest distance between GNSS coordinates. For records without precise coordinates that had information on village administrative unit or protected area, we used the centroid of the boundaries.

## Results

### Age, sex, and physical condition

**Fig 1:**
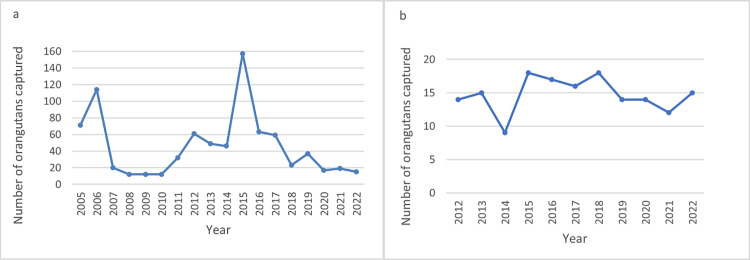
Number of wild orangutans reported captured for translocations: a) in Kalimantan between 2005 and 2022, and b) in Sumatra between 2012 and 2022. No data were available on wild orangutans captured for translocations in Sumatra between 2005 and 2011, hence these years are excluded. Data were collected from published and unpublished sources including newspaper reports, orangutan rescue center news blogs, annual reports, charity commission reports, and social media posts. Any potentially duplicate records were excluded so each capture is only counted once.

We found that at least 988 wild orangutans were captured for translocation in Indonesia between 2005 and 2022 (Fig 1). Sex and estimated age data were available for 693 of these 988 orangutans: 58% (404 individuals) were adults 15 years or older; 43.6% of these adults were female and 53.7% were male, while sex was not reported for 2.7% of the adults (11 individuals). Even elderly animals were captured and translocated: at least 96 individuals (24%) were estimated to be 25 years or older, with the oldest being a 60-year-old male captured and translocated to an unfamiliar habitat. Age data were not exact: 269 individuals were reported simply as belonging to an age category (infant, juvenile, adolescent, or adult), but the years encompassed by these categories were not provided.

Reported translocations included instances where dependent immatures (orangutans six years or younger, generally) were translocated to unfamiliar habitats without their mothers. This was typically because the mothers could not be captured or had been killed, and the immature’s behavior was considered “wild” and thus deemed suitable for translocation. The frequency of such translocations is unknown, due to a lack of data on individuals seen traveling together at the time of capture. Older immature orangutans and adult females traveling together have also been separated by translocations.

Data on the orangutans’ physical condition at time of capture were available for 808 of the 988 individuals captured during the study period. Of these, 660 (81.7%) were recorded as healthy, while 109 (13.5%) were reported to be ill or injured, although in some cases injuries were considered minor and not requiring veterinary care. Twenty (2.5%) orangutans captured were assessed by veterinary personnel as starving or emaciated, and another 19 (2.3%) were considered malnourished to some degree, although it is unclear whether their body condition was outside the normal range of seasonal weight variation.

### Translocation

Our dataset included information on whether the individual was translocated and on the capture and/or release site locations for 268 (27%) of the 988 captured orangutans recorded in our dataset. Of these 268 captured orangutans, only 23.1% (n = 62) were translocated immediately after capture (i.e., without being taken into a captive facility, although generally after being examined in the field by a veterinarian). Nearly half (49.2%, n = 132) were taken into a captive facility for care: 11.9% of the 268 orangutans (n = 32) were in care for less than six months and were subsequently translocated, 33.2% (n = 89) were in captive facilities for at least six months and up to four years prior to translocation, and 4.1% (n = 11) were still in care at the time when data were recorded. Six individuals (2.2%) died following capture, most from human-inflicted injuries sustained prior to capture (n = 4), while for two others a reason was not reported. Data were absent or unclear for 68 of the orangutans (25.4%), and we thus could not confirm their status following capture.

For 79 capture instances involving 86 orangutans (8.7% of the 988 captured), we had sufficient information on both capture and release site locations to analyze distance to release site (S2 Table). Capture and/or release coordinates were missing for 46% of the 86 orangutans, hence for these instances distance was inferred using the centroids from a village administrative unit or a protected area. Most wild translocated orangutans (89.5%, n = 77) were moved more than 10 km from the capture site, while only 5.8% (n = 5) were released within 5 km of their capture location (S2 Table). Nearly a quarter (23.3%, n = 20) were released more than 50 km from their capture location. The longest distance translocations involved moving orangutans to sites outside current species distribution where there are reintroduced populations of orangutans, relocations of 282–706 km from their capture habitats. Median distances in Kalimantan (median = 47.5 km, SD = 25.5 km, max = 98.2 km, n = 28), where orangutan populations are distributed over a larger geographical area, were much longer than those in Sumatra (median = 13.6, SD = 116.3, max = 706.4 km, n = 50).

### Capture habitats and circumstances

Detailed capture records and consultation with practitioners indicated that translocation of wild orangutans generally begins with reports of orangutans seen in or near crops, villages or other infrastructure, or in forest patches or other ‘unexpected’ locations, where authorities or land managers assume they pose a risk of conflict, leading to calls for their removal. The process leading to captures was not detailed for most instances, but we found 99 captures that described local residents or land managers reporting the orangutan to authorities or NGOs.

We found records for 743 captures (75% of all instances in our dataset) with some information on the reason for capture (Table 1). Many captures were associated with multiple confounding circumstances, such as crop foraging that may have been driven by local deforestation, conflict with humans, or forest fires. Data on these potentially confounding factors were rarely available. We recorded the main proximate cause for capture, namely the primary reason someone was alerted to the presence of an animal or asked for it to be removed. For example, if the orangutan was found in a plantation, its presence there was recorded as the reason for capture, regardless of the factors that may have contributed to the animal’s presence in the area.

**Table 1:** Reported reasons for capture of wild orangutans for translocation in Kalimantan and Sumatra, 2005–2022. Information on reason for capture was available for 743 of the total 988 captures. The other 245 captures (26% of 988) are excluded from the table, as reason for capture was unknown. The percentages in the table are of the 743 captures for which relevant data were available. We recorded the primary, proximate reason the animal was captured, namely why someone was alerted to the presence of an orangutan or why they asked for it to be translocated. The underlying reasons for an orangutan’s presence in any particular location can be due to multiple factors such as availability of foods, intraspecific interactions, forest fires, and anthropogenic habitat changes, but lack of available data precludes a comprehensive analysis of these additional drivers.

Primary reason for capture	Number of orangutans captured	% of orangutans captured (n = 743)
**In or near crops and tree plantations**	**513**	**69.0%**
Seen in food crops or tree plantations	218	29.3%
Seen in “area” of crops (reported as near or adjacent to but not in cropland)	247	33.2%
Observed, reported, or assumed crop foraging	48	6.5%
**Seen outside large contiguous forests or arboreal habitats**	**132**	**17.8%**
Seen in a forest patch or other “isolated” area	107	14.4%
Seen in a forest considered unsafe/at risk	22	3.0%
Seen on the ground	3	0.4%
**Seen in or around villages**	**29**	**3.9%**
**Mining and infrastructure**	**11**	**1.4%**
Seen on or near roads	6	0.8%
Seen near power plants, factories, other infrastructure	4	0.5%
Seen in or near coal mining concession	1	0.1%
**Medical or safety interventions**	**30**	**4.0%**
Medical intervention- injured or ill from unknown cause or by conspecifics/other wildlife	13	1.7%
Attacked, restrained, or harassed by humans	17	2.3%
**Forest fires**	**17**	**2.3%**
**Other conflicts**	**5**	**0.7%**
Unspecified conflict with humans	3	0.4%
In conflict with other orangutans	2	0.3%
**Other reasons**	**6**	**0.8%**
Captured by plantation workers or villagers and brought to sanctuary	4	0.5%
Orphaned or assumed orphaned	2	0.3%

Most captures (69% of 743 with relevant data) were initiated as a result of orangutan crop foraging, presence in, or proximity to plantations, gardens, or farms. The second-most common reason was to translocate orangutans considered isolated, stranded, or at risk when seen on the ground or outside of large contiguous forests in smaller fragmented forests, forest patches, or other “isolated” sites (17.8%). Rescue from active forest fires as the primary motive for capture, as opposed to the animal foraging or being reported in crops potentially as a result of forest fires, described 2.3% of captures (Table 1). However, forest fires may be a driver of increased orangutan crop foraging and presence in croplands, villages, and other perceived unsuitable locations; 127 of the 743 captures (17%) mentioned forest fires, even if they were not the primary reason for capture.

**Fig 2:**
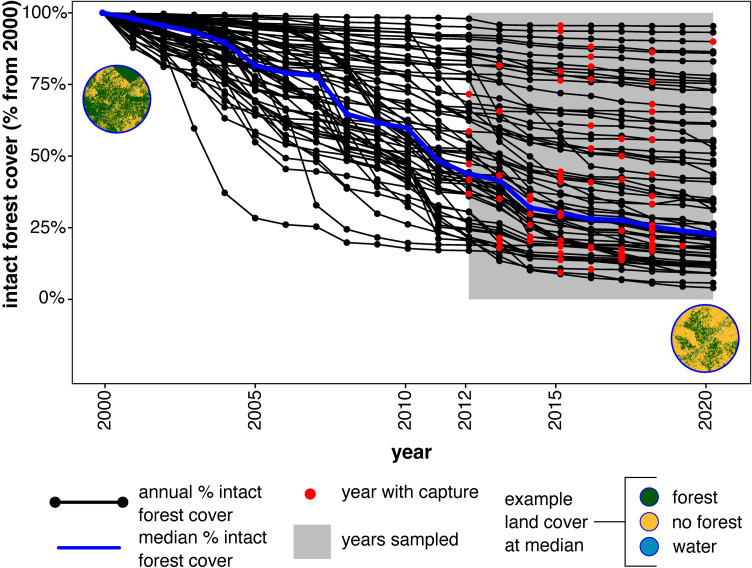
Percentage intact tropical moist forest from 2000 to 2020 around orangutan capture sites. Percentage annual intact tropical moist forest cover (black dots) between 2000 and 2020 was derived from Vancutsem et al. [57]. Annual tropical moist forest layer (at 30 m) and calculated within a 5-km buffer around the capture location (n = 104) in comparison to cover in 2000. Red dots indicate the years in which wild orangutan captures for translocation took place. Grey shading indicates years where capture data is available. Absence of data does not indicate absence of translocation. The blue line is the median percentage forest cover across all capture points (GNSS coordinates). Land cover in the circles represents an example configuration at a location with percentage forest cover in 2020 at the median forest cover. Intact and degraded tropical moist forest and regrowth were combined into a single forest class (green). Remaining areas are either not forested (yellow) or water (blue). Source for forest cover: EC JRC.

Orangutans were captured in or adjacent to natural forests, and in areas 5 km or more from intact forest and forest patches (S3 Table). When previously released orangutans were recaptured, it was often from their original capture site. Analysis of forest cover changes in a 5-km buffer around capture sites for a subsample of 104 orangutans (five in Kalimantan and 99 in Sumatra) with GNSS data showed that the majority were removed from areas that did not have high losses of intact tropical moist forest cover in the year prior to capture (Fig 2). Median intact tropical forest cover loss in the year prior to capture was 3%. Five percent of the sites had intact forest losses of 25% or higher in the year prior to capture. None lost more than 35%. The GLM using forest losses one year prior to capture events was not significantly associated with the likelihood of a capture event compared to the null model including only the intercept (likelihood ratio test, χ2 = 0.031, df = 1, p = 0.86). Conversely, the GLM incorporating losses of intact forests five years prior to capture events was associated with significant likelihood of a capture event in a given year compared to the null model (likelihood ratio test, χ2 = 5.034, df = 1, p = 0.0249), with a slight positive correlation between the percentage of intact forest loss and the probability of a rescue event (coefficient value: 0.01130, p-value: 0.023).

For captures between 2005 and 2017 (n = 53), we assessed forest loss within a 5-km buffer over a five-year period post-capture. The median loss of intact forest across sites was 20% (SD 17%), with individual site losses ranging from 0% to 72%. However, due to data limitations, it was not possible to differentiate this loss from the background rates of deforestation in the study area.

**Fig 3:**
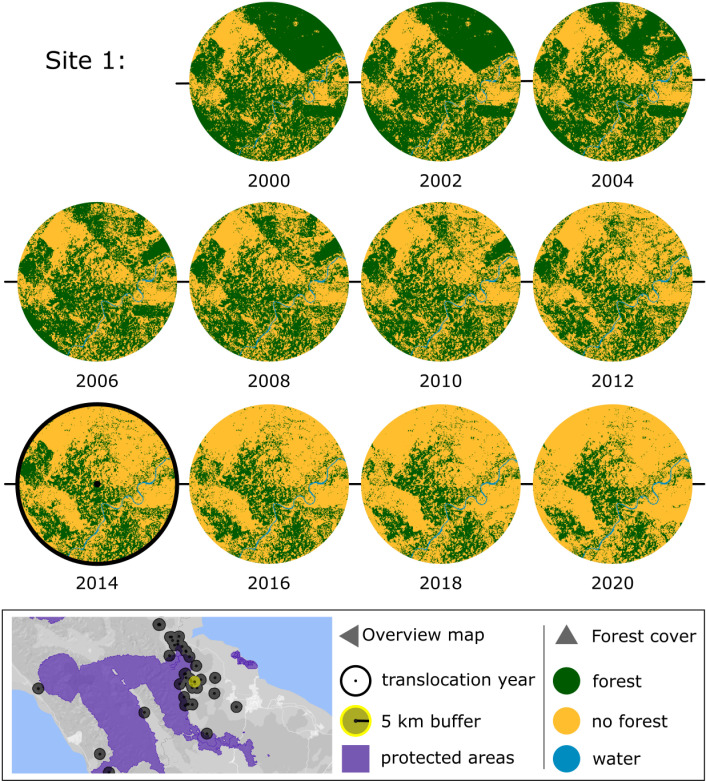
Forest change in area surrounding orangutan capture for translocation site 1. A breeding population of healthy Sumatran orangutans was using a smallholder oil palm and rubber plantation for several years; at the request of the owner to remove seven orangutans, an adult male and a juvenile male were captured and translocated, while an adult female was left on site after unsuccessful capture attempts. Year of capture (2014) is indicated with thick black bordered circle. Intact tropical moist forest, degraded tropical moist forest, and regrowth are combined into a single forest class (green). The map inset at the bottom is the location of the specific capture site and buffer (yellow) along with remaining locations where 97 other orangutans were captured for translocation (black). Basemap: Google Earth Engine. Forest map: EC JRC. Protected areas (purple): UNEP-WCMC and IUCN [60].

**Fig 4:**
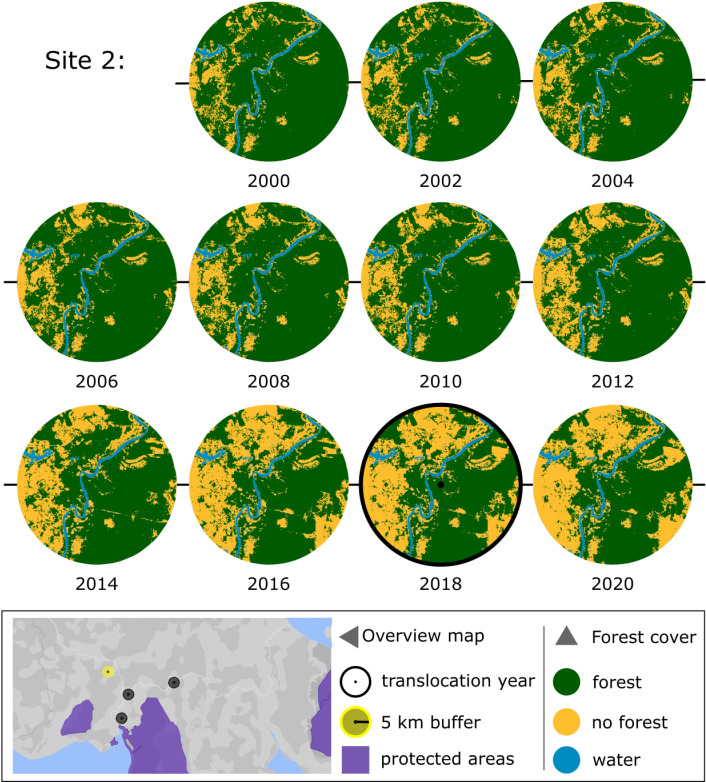
Forest change in area surrounding orangutan capture for translocation site 2. Healthy Bornean adult female and infant that were reported seen in a community rubber plantation and captured for translocation. Year of capture (2018) is indicated with thick black bordered circle. Intact tropical moist forest, degraded tropical moist forest, and regrowth are combined into a single forest class (green). The map inset at the bottom is the location of the specific capture site and buffer (yellow) along with remaining locations where three other orangutans were captured for translocation (black)—one additional orangutan was captured for translocation in an area of Kalimantan outside this map. Basemap: Google Earth Engine. Forest map: EC JRC. Protected areas (purple): UNEP-WCMC and IUCN [60].

To better understand the circumstances of translocations, we reviewed all available information about nine capture sites (five in Sumatra and four in Borneo), and mapped forest cover around these sites using a 5-km buffer (S1 and S2 Figs). Maps of two sites are presented in Figs 3 and 4. At Site 1 (Fig 3), two healthy male Sumatran orangutans, one adult and one juvenile, were captured and translocated. At Site 2 (Fig 4), a healthy Bornean adult female and infant were captured and translocated.

Maps of the remaining seven sites are presented in Supplementary S3–S9 Figs. At Site 3 (S3 Fig), a previously translocated, healthy Tapanuli adult male was recaptured and translocated. At Site 4 (S4 Fig), an injured adult male Sumatran orangutan was captured at the edge of a protected forest. At Site 5 (S5 Fig), a healthy female found traveling with an adolescent was translocated and the adolescent left on site without her; a second healthy adult female was translocated later. At Site 6 (S6 Fig), a seriously injured adult male Tapanuli orangutan was rescued but died shortly thereafter. At Site 7 (S7 Fig), a gravely injured juvenile male Bornean orangutan was rescued but died soon after. At Site 8 (S8 Fig), a healthy adult male Bornean orangutan was captured and translocated for “disturbing” a durian garden. At Site 9 (S9 Fig), a healthy adult male Bornean orangutan reported near a power plant and uninsulated electrical wires was translocated.

Analysis of the nine sites we selected showed the orangutan capture locations were in intact forest, forest patches, degraded forest, or within 5 km of these forests (Figs 3 and 4 and S3–S9 Figs). At six of the nine sites (67%), the orangutans were not at immediate risk from deforestation. In sites 1, 4 and 5, forest within 5 km around translocation site was cleared or degraded two to four years after the orangutans were removed. In sites 4, 6, and 7, the orangutans were at immediate risk of death due to injuries caused by humans (not directly related to forest loss), and no alternative to translocation was immediately available to protect their lives or health.

### Translocation rates

On average, 46 (SD ± 39) orangutans were reported captured annually for translocation in Kalimantan, and 15 in Sumatra (SD ± 3). Annual reported translocation numbers did not show an increasing or declining trend during the study period in Kalimantan (tau = –0.11, 2-sided p value = 0.57) or Sumatra (tau = –0.08, 2-sided p value = 0.81). High variance in Kalimantan translocation numbers compared to Sumatra likely reflects peaks associated with events that impacted Kalimantan habitats, notably forest clearing for agricultural expansion in 2005–2007, and in the record fire years of 2015–2016 (Fig 1). The number of captures reported since 2018 was high in both Kalimantan and Sumatra, with 30–51 animals captured annually in both combined (Fig 1).

The annual number of orangutans removed through the wild-to-wild translocations reported here represented less than 1% of each orangutan species (Table 2). However, annual removals for translocation compound the annual population declines of 3.14% for Bornean (*P. pygmaeus*) orangutans across Kalimantan, and 2.17% for Sumatran (*P. abelii*) and Tapanuli orangutans combined (Table 2). Captured individuals that were taken into captive care prior to translocation or were not translocated (a combined total of approximately 77% of the recorded captures in our dataset) are lost to the wild population at least for the short term. Furthermore, the annual removal rate from wild-to-wild translocation we calculated is likely an underestimate due to probable underreporting of translocation events as well as illegal orangutan killing and capture offtake, which evidence suggests remains high [56,61,62]. Mean estimated killing combined with reported translocation removals was calculated to have removed 3.30% (SD ± 2.61%) of orangutans in Kalimantan, and 11.63% (SD ± 9.49) of orangutans in Sumatra (Table 3). In Kalimantan, this summed estimate approaches the 4% offtake predicted to result in functional species extinction within 75–100 years [51]. Likewise, the summed estimated offtake and translocations in Sumatra exceed the 2% threshold Seaman et al. [51] projected would cause a 76% population decline over 250 years (Table 3, S4 Table).

**Table 2:** Mean annual translocation removals as a percent of population size. Mean annual species and provincial population estimates were calculated from Santika et al. [38], Voigt et al. [19], Utami-Atmoko et al. [43], and Wich et al. [18] (see S4 Table for details). Translocation removals are individuals captured from the wild for translocation but not immediately released into another wild population.

Island	Area/species			Population impacts of removals for translocation 2005–2022				
		Mean current population size^1^	Mean annual % decline^1^	Total reported translocations^2^	Total reported translocation as % of mean population	Mean annual translocations	% Mean population translocated annually	Mean annual decline + translocation removals^3^^,^^4^^,^^5^
Borneo	Borneo (*Pongo pygmaeus*)	83,575	3.14%	829	1.00%	46 (SD ± 39)	0.06%	3.18%
	Central Kalimantan	47,512	2.54%	550	1.16%	31 (SD ± 36)	0.06%	2.58%
	East Kalimantan	14,091	2.05%	176	1.25%	10 (SD ± 9)	0.07%	2.10%
	West Kalimantan	25,079	2.87%	103	0.41%	6 (SD ± 6)	0.03%	2.89%
Sumatra	Sumatra (*P. abelii* & *P. tapanuliensis*)	11,861	2.17%	162	1.37%	15 (SD ± 3)	0.13%	2.27%
	*Pongo abelii* ^6^	11,760	–	155	1.32%	14 (SD ± 3)	0.12%	–
	*Pongo tapanuliensis* ^6^	767	–	7	0.91%	1 (SD ± 1)	0.13%	–

^1^Mean annual population estimates were calculated from Santika et al. [38], Voigt et al. [19], Utami-Atmoko et al. [43], and Wich et al. [18] (see S4 Table for details). Borneo population estimates are from 1999–2019 [19,38,43]. Sumatra population estimates are from 2010–2014 [18].

^2^These numbers are an underestimate of total wild-to-wild translocations as not all translocations are reported, and not all records were available to the authors. Translocations shown for Sumatra are from 2012–2022; no data were available on translocations prior to this range.

^3^Orangutan population estimates are based on available survey data, which are primarily from large metapopulations and protected areas, and modelled based on biophysical and threat predictors of orangutan abundance, and thus are not expected to account for movements of individual orangutans through wild-to-wild translocation.

^4^Over time, annual offtake and translocation removals of more than 1% in additional to natural mortality is expected to drive orangutan populations to extinction in suboptimal habitats [52], and is predicted to result in approximately 75% population decline at 2% or higher and to lead to functional extinction of populations at 4% or higher [51].

^5^% Mean population translocated was reduced by 23% to account for the estimated percentage of individuals immediately released into another wild population (S4 Table) and assumed to survive, and thus not considered removed from the area/species population.

^6^We did not calculate mean annual percent declines for Sumatran and Tapanuli orangutan species because published species-specific population numbers since 2016 were not available at time of writing.

**Table 3:** Impact of reported translocation removals and estimated killing offtake. Orangutan killing estimates are calculated from the mean, minimum, and maximum rates in Meijaard et al. [61] and crime detection rates of 1.2%–10% from Sherman et al. [56].

Island	Area/species	Population size	Total trans-locations reported	Annual translocations(%)^3^	Killing estimates	Annual killing offtake (%)	Killing and trans-location removals (%)^6^	Source
Borneo	Kalimantan (*P. pygmaeus*)	83,575^1^	829	46 (0.06%)^4^	Mean annual	2,540 (3.04%)	3.08%	Meijaard et al. 2011
					Minimum annual	2,383 (2.85%)	2.89%	
					Maximum annual	3,882 (4.64%)	4.68%	
					Minimum annual over lifetime	630 (0.75%)	0.80%	
					Maximum annual over lifetime	1,357 (1.62%)	1.67%	
					Mean at 1.2% detection rate	8,232 (9.85%)	9.90%	Sherman et al. 2022
					Mean at 3.2% detection rate	3,084 (3.69%)	3.74%	
					Mean at 6.2% detection rate	1,387 (1.66%)	1.71%	
					Mean at 10% detection rate	986 (1.18%)	1.23%	
Sumatra	*P. abelii* & *P. tapanuliensis*	11,861^2^	162	15 (0.13%)^5^	Mean at 1.2% detection rate	3,250 (27.40%)	27.45%	Sherman et al. 2022
					Mean at 3.2% detection rate	1,222 (10.30%)	10.35%	
					Mean at 6.2% detection rate	629 (5.30%)	5.35%	
					Mean at 10% detection rate	391 (3.30%)	3.35%	

^1^Mean annual population estimates for Borneo population for 1999–2019 were calculated from Santika et al. [38], Voigt et al. [19], Utami-Atmoko et al. [43], and Wich et al. [18] (see S4 Table for details).

^2^Mean annual population estimates for Sumatra for 2010–2014 were calculated from Wich et al. [18] (see S4 Table for details).

^3^Numbers are an underestimate of total wild-to-wild translocations as not all translocations are reported, and not all records were available to the authors.

^4^Translocations shown for Borneo are from 2005–2022. Mean annual translocation removal is equal to the total reported translocations/18 years.

^5^Translocations shown for Sumatra are from 2012–2022; no data were available on translocations prior to this range. Mean annual translocation removals is equal to total reported translocations/11 years.

^6^% Contribution of translocation removals was reduced by 23% to account for the estimated percentage of individuals immediately released into another wild population (S4 Table) and assumed to survive, and thus not considered removed from the area/species population.

These estimated overall offtake and translocation removal rates are for the entire orangutan populations in Indonesian Borneo and Sumatra, but as the threats and rates of population decline vary among specific populations [63], the percentage for some populations would be much higher than the average shown here. Translocation removals did appear to pose higher risks in small or dispersed populations and may affect metapopulation function through the removal of breeding age individuals from local populations. Translocation rates appeared to pose a particularly high risk to the Tapanuli species, given their small population size. Tapanuli orangutans occupy a small, relic range [64] with considerable anthropogenic habitat changes and increasing human-orangutan conflicts [65]. The average of one animal per year removed through translocation during the study period represented 0.13% of the total estimated remaining species population (Table 2). Similarly, translocation removals appear to have more marked effects on dispersed populations and subpopulations for all species. A subsample of 528 captures with information on the village administrative unit (*desa*) location showed 11 *desa* (three in Sumatra and eight in Kalimantan) that each had at least seven orangutans captured and removed during the 2005–2022 study period (S5 Table). Six of these *desa* had 15 or more individuals captured for translocation, the majority of which were adult (breeding age) females and males (S5 Table). The largest translocation was 106 individuals removed from a single *desa* in Kalimantan, with 105 of them removed over a four-year period. The rationale for removing what was effectively an entire population of 105 individuals was that the habitat was fragmented and there were risks of further fragmentation and fire. In this *desa* (Tumbang Mangkutup, S5 Table) 14,055 ha of forest (43%) were lost in the year following removal of 76 orangutans in 2015 per Nusantara Atlas [66]. Forest losses in the year preceding the translocations totaled 695 ha, and were minimal in the years after 2016 (94 ha in 2018, and zero or a few hectares in all other years after 2016) [66].

Many reproductively valuable males and resident breeding females have been translocated away from fragmented forests and forest patches in unprotected areas and Figs 3 and 4 and S3–S9 Figs indicate that these translocations have broken up dispersed genetic clusters. Some areas may have been largely emptied of orangutans in response to repeated requests from communities, industry or government agencies for animals to be removed from their properties or crops (see S5 Table).

### Post-release survival

Translocated wild orangutans were rarely monitored, either not at all or only for a few days to a week post-release, due to the practical difficulties of following them. They are not typically given radio-tracking implants, which require surgery and recovery time in a medical facility [67]. Consequently, only one small sample of survival data was available, which showed that for 21 individuals released over a two-year period, 14 orangutans were encountered over the course of 10 monthly monitoring surveys. This information is difficult to interpret as it does not include information on presence or population size of conspecifics in the release area, and it is not known how long after release any of the individuals were sighted. Communications with practitioners during this study did not yield any further evidence on survival of released individuals or overall survival rates across the wild-to-wild translocations they conducted. Thus, overall survival rate of orangutans wild-to-wild translocated in Indonesia since 2005 is unknown.

### Welfare outcomes

Videos and written reports of captures for translocation show orangutans that were being pursued displaying signs of extreme stress, including fleeing, screaming, and ultimately defensive aggression towards humans when approached and during capture. One captured orangutan was reported to have a dangerously high body temperature. Although this individual appeared to recover, it is not known how he fared after translocation. One set of records on 166 orangutans wild-to-wild translocated from oil palm plantations shows that 19 (11%) died during or shortly after capture, although at least one orangutan that died had been previously injured by humans. A few reported cases described orangutans being chased and darted with anaesthetic and then ultimately not captured and translocated, although we do not know how common this is. Welfare risks to the animals increase if they are already in physical stress (such as being malnourished, dehydrated, or pregnant, all of which have been recorded in captured orangutans), and with each capture or recapture attempt and each translocation.

Wild-to-wild translocated orangutans were recorded returning to the areas they were captured from or traveling to other areas with human food crops. Overall prevalence of recaptures is not known due to lack of consistent data on identity of captured individuals, but a study analyzing data from one year (March 15, 2020–March 14, 2021) showed that 31% of the 26 wild-to-wild orangutan translocations recorded were recaptures of previously captured and translocated orangutans [11]. This includes a female Sumatran orangutan who was captured and translocated at least five times [68], and a flanged male Tapanuli orangutan captured and translocated twice [69,70].

## Discussion

Data on wild-to-wild translocation of orangutans in Indonesia between 2005 and 2022 highlight the urgent need to carefully weigh the perceived necessity for each capture and translocation, as they likely have deleterious effects on orangutan populations and metapopulations, and often on individual orangutans. The probability of the individuals and populations surviving in their original habitat and in identified suitable release habitats should be carefully assessed, with focus on preventing risks to existing wild populations and genetic flow. Whenever wild-to-wild translocation is deemed necessary, it is critical that data on post-release outcomes are collected.

### Age, sex and health status

Adult males (15 years or older) were the most commonly translocated age and sex class in our dataset. The dispersal and ranging patterns of adult males make them more likely to travel into areas that are less familiar to them and to encounter risks of both conflict with humans and being captured for translocation during their travels. Adult male orangutans exhibit bi-maturism with two distinct developmental phases: they are able to reproduce successfully in both phases, but in the unflanged phase they lack the full complement of the secondary sexual development of the flanged phase, characterized by prominent cheek pads (flanges), increased body size, and a larger laryngeal sac [71]. It is natural behavior for unflanged and flanged males to range widely across the landscape, following fruiting cycles and seeking reproductive opportunities, and at times avoiding competition with other males [37,72,73]. Because flanged males are aggressive towards each other [73], translocating them puts them and any resident flanged males at a sudden increased risk of aggression, injury, or death after release. Translocated unflanged males will likely move away from the release area and may return to the area they were removed from or may enter other croplands.

Adult females were the second most commonly translocated age and sex class (n = 176), despite being known to have strong attachment to the sites where they live, and being intolerant of unrelated females [26,27,29,74]. Capturing and moving females to unfamiliar habitats is likely to provoke resident females to be aggressive towards them and potentially exclude them from some food resources [27,75]. During periods of low food availability, orangutans have lower tolerance for each other, even for female kin [76]. This could generate significant social stress and render translocated females unable to access adequate food.

Although less common, wild-to-wild translocations that separate dependent infants or immatures traveling with an adult female are a particular concern. Regardless of whether infant orangutans display abilities or behaviors appropriate for wild orangutans, they do not range independently until they are approximately eight years or older [29]. Immature adolescent orangutans below 15 years old travelling with adult females are usually older offspring who continue to range alongside their mother in her matrilineal range while building their ability to range and forage independently [37,77]. Separating and translocating these immature orangutans from their mother, even if they are eight years old or more and traveling separately, means they may not be able to survive or thrive without her.

Evidence from this study does not support the frequently reported claim that adult orangutans seen in plantations or cultivated crops are generally starving or malnourished and in need of rescue, even when they appear to be underweight [5]. Orangutans’ body weight and condition fluctuate with food availability, and in lean times they lose weight and even catabolize muscle [78]. If translocated, they will almost certainly have a harder time finding sufficient food than if left in habitat they know, particularly if moved to an unfamiliar forest type [79,80] and if they have to compete with resident orangutans. Weakened body condition can make them more vulnerable to disease, and translocating malnourished orangutans would cause them additional strain, further lessening their chances for post-release survival, particularly if they have to find food in an unfamiliar habitat in addition to competing with resident orangutans. Healthy but malnourished orangutans are best left to continue foraging in the area they already know, unless it is physically impossible for them to find sufficient food—for example, if no food sources remain in the vicinity. Orangutans are known to travel at least five kilometers into plantations and other modified landscapes [M. Ancrenaz, unpublished data; [35,44,51].

### Capture habitats and circumstances

Large- and small-scale deforestation in forested orangutan strongholds, clearing of forest patches and large trees in fragmented forests and agricultural landscapes, and clearing for mining, hydropower and infrastructure have collectively resulted in marked reductions in forested orangutan habitats [17,47,81]. These changes fragment orangutan populations, leaving females residing in dispersed forest patches left after clearing, and males traveling between these patches [37,44]. These “isolated” orangutans are often assumed to be lost or stranded and doomed to starve unless translocated. Due to lack of empirical evidence on the threats to the orangutan at the capture site and on any interventions made to ameliorate those threats and protect the individual in situ, it was not possible to statistically assess what percentage of the captured orangutans in our dataset would have been in mortal danger had they not been moved. However, mapping of orangutan capture sites indicated that most translocated orangutans could have returned to larger forests on their own, moved to other areas (as is normal behavior for males), or continued living where they were (as is normal behavior for adult females) if undisturbed by humans and if remaining tree cover, including small patches, is left standing. This suggests that many translocations are not directly related to deforestation levels that would be life threatening for orangutans. This is also supported by the lack of significant correlation between recent deforestation and capture likelihood. The positive association we found between likelihood of a capture event and deforestation in the area five years prior could be influenced by increased crop foraging as orangutans modify their foraging habits to accommodate these habitat changes. This also may explain why deforestation one year prior was not significantly associated with capture likelihood—it takes some time for orangutans to start using new types of landscapes (Ancrenaz, unpublished data). Over time we expect to see an increasing number of orangutans foraging or traveling within these multiple-use landscapes. The data also show that deforestation in orangutan habitats is ongoing, which reduces native food availability and drives ever more overlap between orangutans and humans. This underscores the urgency to rethink how wild-to-wild translocation is used: as more orangutans are encountered in plantations, gardens, and multiple-use landscapes across their ranges, using translocation as a default management approach risks widespread negative impacts on local populations and metapopulations.

### Conservation and welfare outcomes

Translocation is not a long-term solution to address human-orangutan conflicts or to assuage people’s concerns over orangutan sightings. Nonetheless, approximately 20% of translocation practitioners we communicated with reported that government authorities required them to move orangutans as the first intervention, rather than attempt to sensitize people to orangutan behaviors and develop strategies to protect the animals in place. Most people who report orangutan sightings want them to be removed [5]. To stem the number of risky translocations and prevent negative human-orangutan interactions, it is critical to immediately invest across the orangutan range in simultaneously building realistic expectations of orangutan behavior and deploying strategies that ease immediate risks and inconveniences to humans (e.g., by creating financial benefits from coexistence with orangutans, or other compensatory benefits such as jobs or local services), while also protecting orangutans in situ.

Regardless of perceptions of translocation, orangutans will continue to find their way to appetizing crops even after they have been translocated to other sites. Removing all orangutans that visit crops or human-use areas will result in emptying the metapopulation of resident female clusters and ranging males, dramatically diminishing the genetic fitness and long-term viability of the three species. Furthermore, it would not be logistically and financially feasible to translocate the tens of thousands of orangutans that overlap with human-used lands or live near humans [82]. Releasing so many individuals into existing protected areas with resident orangutan populations, as is commonly happening [5], would cause extreme social disruption and likely result in increased competition for food resources, poorer nutrition, increased social stress, lowered birth rates, poor welfare, and increased mortality for both translocated and resident orangutans.

Orangutan socioecology, and instances of translocated orangutans found killed [10] or recaptured [11] suggest that compromised survival and welfare of released individuals may be a common outcome. Although released orangutans are rarely found dead, this absence of data does not necessarily indicate a high likelihood of survival. Few animals are found dead in tropical forests [83]. Orangutan researchers report that dead orangutans are rarely encountered, even in sites where they are followed daily and studied intensively for decades (Wich, pers. obs.). Furthermore, orangutans are in some cases translocated into remote areas where it is even less likely their bodies would be detected.

Although preventing human-orangutan conflicts and reducing the potential for human killing or injuring of orangutans are common goals of wild-to-wild translocations, it is unclear how well they serve this function as currently applied. Translocation may avoid further injury and eventual death in cases where orangutans are attacked by humans at the capture site, but since translocated animals are rarely monitored, the survival and welfare of the released individuals are mostly unknown. Practitioners commonly noted that people threaten to kill orangutans that crop forage, but often orangutans reported by residents or land managers cannot be found by translocation teams and are thus left on site by default. However, in one published example, one wild-to-wild translocated male was killed after returning to the plantation where he was originally captured [10]. Thus, wild-to-wild translocations do not necessarily prevent orangutans from being killed or injured by humans. At least 61 (8%) of the 797 orangutans with health data in our dataset had already been harmed by humans at some point, generally with air rifle bullets, and many had multiple recent bullet wounds.

More than 227 wild-to-wild translocated orangutans have been released into sites with viable orangutan populations that do not need supplementation, including Tanjung Puting, Gunung Palung, Kutai, and Gunung Leuser National Parks and the Mawas conservation area [43], where their release could provoke social stress and competition for natural resources, and pose disease transmission and genetic risks to resident orangutans [11]. In Sumatra, the prevalence of multiple captures in small areas are likely to negatively impact local populations and increase fragmentation of the orangutan metapopulations in these locations (S2 Fig).

Sustaining viable metapopulations of orangutans across large landscapes will require engaging local communities and land managers to preserve the remaining forests (including forest fragments) and protect orangutans without resorting to translocation [42,44,84]. We encourage those living or operating in orangutan range to adopt policies following IUCN SSC Primate Specialist Group guidelines for orangutan wild-to-wild translocation (Sherman et al., in press [85]). These guidelines follow the precautionary principle, stipulating that where there is insufficient evidence to demonstrate that an action will not harm an individual orangutan’s welfare or the conservation of the species, it must then be assumed this action could cause harm, and the action should thus be avoided.

### Recommendations

We recommend an ‘Identify, Monitor, and Protect’ approach, explained below, focusing on preventing conflicts and avoiding interventions with wild orangutans except in extraordinary circumstances.

#### Identify risk level.

The first step in cases of habitat encroachment or clearing is to check and enforce any laws and local policies protecting orangutans and orangutan habitat. For areas already impacted by clearing or other human activities, map habitat and forest cover for at least 5 km surrounding the orangutan’s location to determine if it could move away on its own. Updated, detailed information on habitat and forest cover can be obtained free of charge from satellite-derived landcover data (e.g., UM and WRI [86]). If the immediate area is degraded or cleared, but there are forest blocks (large or small) or forest corridors within 5 km, translocation is not necessary. Be sure that human activity including monitoring is not so invasive that it restricts the orangutan’s options and ability to move away without intervention.

People or companies actively trying to kill, injure, harass, drive away, or capture orangutans, should first be apprised of the illegality of these actions. Orangutans are fully legally protected in Indonesia and Malaysia, and harm, killing, or capture of orangutans is punishable under existing laws. Governments have the authority, and the responsibility, to enforce these laws if businesses and/or individuals are unwilling to comply. Providing alternative approaches, such as working with relevant parties to preserve natural forest habitat and to find non-lethal ways to address the conflict without translocation will also be vital. Orangutans at risk of being harmed or killed should be monitored from a safe distance (≥50 meters) to discourage defensive action by the animal or further stress or harm to the individuals.

#### Monitor orangutans potentially at risk.

Orangutans appearing underweight or with suspected human injuries should be monitored from ≥ 50 m for at least five days before any decision is made. Keep humans and dogs away, and do not surround the orangutan on all sides. It is often best, especially if the orangutan has no shelter, to leave the area and return to monitor periodically. If the orangutan moves and feeds within the five days, no intervention is needed. Orangutans with serious, human-inflicted injuries should be transferred to specialized rehabilitation centers.

#### Protect transient and resident orangutans.

There are many actions that can be proactively taken to protect orangutans in the spaces they use. The following actions are recommended for orangutan stakeholder communities and sectors:

Landowners and managers and in situ conservation projects can protect orangutans in place by preserving both large intact forests and forest fragments of any size in mixed-use landscapes. Building over-passes and/or aerial bridges for existing roads and other infrastructure will allow safe movement by orangutans. Orangutan rescue centers and conservation groups, in collaboration with social scientists, can conduct outreach designed to improve local understanding about orangutans foraging on cultivated crops even where large forests are available. Rescue centers, businesses, and government can collaborate with local communities to develop suitable coexistence strategies involving regular on-the-ground monitoring and developing understanding and, if necessary, incentives for communities to accept living alongside orangutans, and for local wildlife protection agencies to promote coexistence rather than translocation.

Individual orangutans can be wild-to-wild translocated responsibly when: 1) they are suffering from a serious human-caused injury and have been successfully treated by a veterinarian, or, 2) they have no path to escape from a severe threat in their current location, such as fire or flooding. With the recognition that these situations are indicative of deficits in conservation management, wild-to-wild translocation may be necessary for the following additional welfare reasons: 1) people are trying to harass, capture, or kill an orangutan despite attempts to enforce protection laws, 2) interventions to halt habitat clearing have failed and an orangutan has no path to escape, and 3) orangutan specialists have confirmed reports of unprovoked orangutan attacks on people. In the cases outlined above, orangutans should be translocated to a nearby well-protected forest area, and, if possible, into a community of related orangutans, and monitored post-release.

Wild-to-wild translocation is not suitable for addressing threats to multiple individuals living in the same area, to enable or offset clearing orangutan habitats, or to remove wild orangutans who are merely crossing human-use areas or visiting cultivated crops adjacent to or very near a forest (a few kilometers or less). The priority for effective species conservation is to preserve remaining habitat and protect both resident and transient orangutans in situ. Translocating populations to enable clearing or prevent crop foraging is contrary to the precautionary principle, violates existing laws that protect these Critically Endangered species, reduces natural habitat remaining, and diminishes species recovery potential.

Wild-to-wild translocation is not a long-term solution to most human-wildlife conflicts [8], and can pose serious risks to the conservation of rare species. The approach outlined here would reduce use of high-risk interventions and promote long-term solutions for human-orangutan coexistence. It could be adapted for application to other species, especially wide-ranging species that are threatened by habitat fragmentation and the potential for interactions with humans in mixed use landscapes. It may become particularly relevant for the African great apes: although wild-to-wild translocation of African apes is extremely rare as of now, increased clearing and fragmentation of their habitats may drive demand for translocation as a means to facilitate development while supposedly “saving” great apes that are ‘in the way’ of planned land uses. However, wild-to-wild translocation could be even more problematic for African apes, which have more complex, less diffuse group structures than orangutans (see for example: [87]). It would not serve to resolve underlying conflicts and would involve risks to resident conspecific and sympatric apes that would violate the precautionary principle for great ape translocations.

## Supporting information

S1 Appendix Data tables on orangutan captures for translocation in Indonesia, 2005 to 2022. (DOCX)

S1 Table List of data sources. Online links were last accessed on December 16, 2024 unless otherwise noted. Searches were limited to the date range of January 1, 2005–December 31, 2022. Some data sources could not be listed due to confidentiality issues. The table does not list webpage links for previously public sources that are no longer available online. We used additional publicly available and unpublished data from individuals and organizations not listed here due to confidentiality concerns.
(DOCX)

S2 Table Distance from capture site to release area. Data were available for 86 captured and translocated orangutans. Locations were measured as straight line shortest distance between GNSS coordinates, or, where coordinates were not available, from the centroid of the capture village administrative unit (*desa*) and the centroid of the release area (protected area shape file or *desa*).
(DOCX)

S3 Table Habitat types and crops where orangutans were encountered in Kalimantan and Sumatra, 2005–2022. Sources: Records of captures for translocation (this study), published literature, and communications with orangutan researchers and translocation practitioners.
(DOCX)

S4 Table Orangutan population estimates and mean annual rate of decline. Population figures are based on most recent published estimates in the listed sources.
(DOCX)

S5 Table Village administrative units (*desa*) where seven or more orangutans were reported as captured for wild-to-wild translocation between 2005 and 2022 (Kalimantan) and 2012 to 2022 (Sumatra). Data on *desa* locations were available for 528 of the 988 reported captures during the study period. F = female, M = male. Estimated age classes are: infant (I) 0–6 years, juvenile/adolescent (J) 7–12 years, adult (A) 15 or more years, and unknown (U). Unknown sex or age means these data were not available in the records we reviewed. Age and sex data may have been collected but not made available in the records we were able to access.
(DOCX)

S1 Fig Overview of land cover changes around orangutan capture sites in Sumatra, 2000–2022. Depicted areas cover a 5-km buffer around GNSS coordinates of sites where 99 orangutans were captured for translocation between 2000 and 2022. Intact tropical moist forest, degraded tropical moist forest, and regrowth from Vancutsem et al. [57] were combined into a single forest class (green) to visualize forested areas available to orangutans. Several captures were reported within the same areas and thus have overlapping buffers. Protected areas (purple): UNEP-WCMC and IUCN [60]. Forest map: EC JRC. Basemap: Google Earth Engine.
(TIF)

S2 Fig Overview of land cover changes around orangutan capture sites in Kalimantan. Land cover changes are shown for a 5-km buffer around GNSS coordinates of four sites where five orangutans were captured for translocation between 2000 and 2022. Top panels show one capture site in East Kalimantan. Bottom panels show three capture sites in Central Kalimantan. Intact tropical moist forest, degraded tropical moist forest, and regrowth from Vancutsem et al. [57] were combined into a single forest class (green) to visualize forested areas available to orangutans. Protected areas (purple): UNEP-WCMC and IUCN [60]. Forest map: EC JRC. Basemap: Google Earth Engine.
(TIF)

S3 Fig Forest change in area surrounding orangutan capture for translocation site 2. Recapture of a healthy Tapanuli adult male originally captured and translocated in 2019. He had returned to the original capture area and was seen alongside a road. Year of capture (2020) is indicated with a thick black bordered circle. Intact tropical moist forest, degraded tropical moist forest, and regrowth are combined into a single forest class (green). The map inset at the bottom is the location of the specific capture site and buffer (yellow) along with remaining locations where 1 other orangutan was captured for translocation (black). Basemap: Google Earth Engine. Forest map: EC JRC. Protected areas (purple): UNEP-WCMC and IUCN [60].
(TIF)

S4 Fig Forest change in areas surrounding orangutan capture for translocation in site 4. An injured Sumatran adult male reported as seasonally foraging in durian (*Durio* sp.) and langsat (*Lansium parasiticum*) gardens at the edge of protected forest. Year of capture (2018) is indicated with thick black bordered circle. Intact tropical moist forest, degraded tropical moist forest, and regrowth are combined into a single forest class (green). The map inset at the bottom is the location of the specific capture site and buffer (yellow) along with remaining locations where 98 other orangutans were captured for translocation (black). Basemap: Google Earth Engine. Forest map: EC JRC. Protected areas (purple): UNEP-WCMC and IUCN [60].
(TIF)

S5 Fig Forest change in area surrounding orangutan capture for translocation in site 5. A Sumatran orangutan population reported as isolated in area considered at risk for deforestation; two healthy adult females were translocated from this site. Year of capture (2013) is indicated with thick black bordered circle. Intact tropical moist forest, degraded tropical moist forest, and regrowth are combined into a single forest class (green). The map inset at the bottom is the location of the specific capture site and buffer (yellow) along with remaining locations where 97 other orangutans were captured for translocation (black). Basemap: Google Earth Engine. Forest map: EC JRC. Protected areas (purple): UNEP-WCMC and IUCN [60].
(TIF)

S6 Fig Forest change in area surrounding orangutan capture for translocation in site 6. An injured Tapanuli adult male in a salak fruit (*Salacca zalacca*) plantation. Year of capture (2013) is indicated with thick black bordered circle. Intact tropical moist forest, degraded tropical moist forest, and regrowth are combined into a single forest class (green). The map inset at the bottom is the location of the specific capture site and buffer (yellow) along with remaining locations where 1 other orangutan was captured for translocation (black). Basemap: Google Earth Engine. Forest map: EC JRC. Protected areas (purple): UNEP-WCMC and IUCN [60].
(TIF)

S7 Fig Forest change in area surrounding an orangutan capture for translocation in site 7. A gravely injured juvenile male Bornean orangutan, previously thought to have been foraging in a local pineapple garden within Kutai National Park. Year of capture (2018) is indicated with thick black bordered circle. Intact tropical moist forest, degraded tropical moist forest, and regrowth are combined into a single forest class (green). The map inset at the bottom is the location of the specific capture site and buffer (yellow). Basemap: Google Earth Engine. Forest map: EC JRC. Protected areas (purple): UNEP-WCMC and IUCN [60].
(TIF)

S8 Fig Forest change in area surrounding orangutan capture for translocation site 8. Healthy Bornean adult male reported to be staying in and “disturbing” a local durian garden. Year of capture (2019) is indicated with thick black bordered circle. Intact tropical moist forest, degraded tropical moist forest, and regrowth are combined into a single forest class (green). The map inset at the bottom is the location of the specific capture site and buffer (yellow) along with remaining locations where three other orangutans were captured for translocation (black). Basemap: Google Earth Engine. Forest map: EC JRC. Protected areas (purple): UNEP-WCMC and IUCN [60].
(TIF)

S9 Fig Forest change in area surrounding orangutan capture for translocation site 9. Healthy Bornean adult male seen in APL (non-forest) land near a power plant and uninsulated electrical wires. Year of capture (2018) is indicated with thick black bordered circle. Intact tropical moist forest, degraded tropical moist forest, and regrowth are combined into a single forest class (green). The map inset at the bottom is the location of the specific capture site and buffer (yellow) along with remaining locations where three other orangutans were captured for translocation (black). Basemap: Google Earth Engine. Forest map: EC JRC. Protected areas (purple): UNEP-WCMC and IUCN [60].
(TIF)
