# Supplementary material for: Outcomes of orangutan wild-to-wild translocations reveal conservation and welfare risks
Source: PLoS One. 2025 Mar 19;20(3):e0317862. doi: 10.1371/journal.pone.0317862 (PMC11970725; doi:10.1371/journal.pone.0317862)
Supplement: S1 Appendix — (DOCX) [file pone.0317862.s001.docx]

**S1. Appendix. Data tables on orangutan captures for translocation in Indonesia, 2005 to 2022.**

**Table A. Annual captures for translocation in Kalimantan, 2005 – 2022.**

| 2005 | **71** |
| --- | --- |
| 2006 | **114** |
| 2007 | **20** |
| 2008 | **12** |
| 2009 | **12** |
| 2010 | **12** |
| 2011 | **32** |
| 2012 | **58** |
| 2013 | **49** |
| 2014 | **46** |
| 2015 | **157** |
| 2016 | **63** |
| 2017 | **68** |
| 2018 | **24** |
| 2019 | **37** |
| 2020 | **17** |
| 2021 | **19** |
| 2022 | **15** |

**Table B. Annual captures for translocation in Sumatra, 2012 – 2022.**

| 2012 | 14 |
| --- | --- |
| 2013 | 15 |
| 2014 | 9 |
| 2015 | 18 |
| 2016 | 17 |
| 2017 | 16 |
| 2018 | 18 |
| 2019 | 14 |
| 2020 | 14 |
| 2021 | 12 |
| 2022 | 15 |

**Table C. Sex and age class of orangutans captured for translocation in Indonesia, 2005 – 2022.**

|  | **Female** | **Male** | **Unknown sex** |
| --- | --- | --- | --- |
| **Reported age class or age in years**^1^ | | | |
| Infant | 19 | 19 | 24 |
| Juvenile | 7 | 9 | 2 |
| Adolescent | 3 | 4 |  |
| Subadult |  | 2 |  |
| Adult | 76 | 90 | 11 |
| 0.1 or less | 1 |  | 2 |
| 0.3 |  | 2 | 1 |
| 0.5 | 1 |  |  |
| 0.8 |  |  | 1 |
| 1 | 5 | 5 |  |
| 1.5 | 3 | 3 |  |
| 2 | 2 | 13 | 1 |
| 2.5 | 2 | 3 |  |
| 3 | 4 | 3 | 1 |
| 3.5 | 1 | 1 |  |
| 4 | 9 | 6 | 1 |
| 4.5 | 1 |  |  |
| 5 | 6 | 1 | 1 |
| 6 | 8 | 6 |  |
| 7 | 10 | 4 |  |
| 7.5 | 1 | 1 |  |
| 8 | 4 | 2 |  |
| 9 | 6 | 1 |  |
| 10 | 11 | 10 |  |
| 11 | 7 | 1 |  |
| 12 | 14 | 6 |  |
| 12.5 | 1 | 1 |  |
| 13 | 12 | 2 |  |
| 14 | 10 | 2 |  |
| 15 | 30 | 22 |  |
| 16 | 11 | 5 |  |
| 17 | 1 | 6 |  |
| 18 | 3 | 1 |  |
| 19 |  | 2 |  |
| 20 | 17 | 23 |  |
| 21 |  | 1 |  |
| 22 | 1 | 2 |  |
| 23 |  | 2 |  |
| 24 | 1 | 3 |  |
| 25 | 14 | 21 |  |
| 26 | 1 | 4 |  |
| 28 | 2 | 1 |  |
| 30 | 14 | 22 |  |
| 31 |  | 2 |  |
| 35 | 4 | 8 |  |
| 38 | 1 |  |  |
| 40 |  | 1 |  |
| 60 |  | 1 |  |
| Unknown age | 20 | 14 | 261 |
| **Total** | **344** | **338** | **306** |

1. Records with a reported age class did not define years included in the class.

**Table D. Reported condition of orangutans captured for translocation in Indonesia, 2005 – 2022.** Data on condition were available or extractable from records on 808 of 988 orangutans. Individuals were assumed healthy unless otherwise noted.

| **Condition** | **Number of orangutans** | **Percent** |
| --- | --- | --- |
| Starving or emaciated | 20 | 2% |
| Malnourished or dehydrated | 19 | 2% |
| Healthy | 660 | 82% |
| Ill or injured | 109 | 13% |

**Table E. Reported village (*desa*) location of orangutans captured for translocation in Indonesia, 2005 – 2021.** None of the records for 2022 captures included village locations.

|  | **Number of orangutans captures per year** | | | | | | | | | | | | | | | | | |
| --- | --- | --- | --- | --- | --- | --- | --- | --- | --- | --- | --- | --- | --- | --- | --- | --- | --- | --- |
| **Province**  *Village* | **2005** | **2006** | **2007** | **2008** | **2009** | **2010** | **2011** | **2012** | **2013** | **2014** | **2015** | **2016** | **2017** | **2018** | **2019** | **2020** | **2021** |  |
| **Aceh** | | | | | | | | | | | | | | | | | | |
| *Air Pinang* |  |  |  |  |  |  |  |  |  |  |  |  |  |  | 1 |  |  |  |
| *Alur Kejrun* |  |  |  |  |  |  |  |  |  |  |  |  |  | 1 |  |  |  |  |
| *Alur Selelas* |  |  |  |  |  |  |  |  |  |  |  |  |  |  | 2 |  |  |  |
| *Bandar Setia* |  |  |  |  |  |  |  |  |  |  |  |  |  | 3 |  |  |  |  |
| *Beusa Baroh* |  |  |  |  |  |  |  |  |  |  |  |  |  | 1 |  |  |  |  |
| *Blang Makmur* |  |  |  |  |  |  |  |  |  |  |  |  |  |  | 1 |  |  |  |
| *Bunga Tanjung* |  |  |  |  |  |  |  |  |  |  |  |  |  |  | 2 |  |  |  |
| *Gampong Tengah* |  |  |  |  |  |  |  |  |  |  |  |  |  |  | 1 |  |  |  |
| *Harum Sari* |  |  |  |  |  |  |  |  |  |  |  |  |  | 1 |  |  |  |  |
| *Hutan* |  |  |  |  |  |  |  | 1 |  |  |  |  |  |  |  |  |  |  |
| *Hutan Tangantangan* |  |  |  |  |  |  |  |  |  |  |  |  |  | 1 |  |  |  |  |
| *Ie Merah* |  |  |  |  |  |  |  |  |  |  | 1 |  |  |  |  |  |  |  |
| *Jambo Dalem* |  |  |  |  |  |  |  |  |  |  |  |  | 1 |  |  |  |  |  |
| *Kapa Seusak* |  |  |  |  |  |  |  |  |  |  |  |  |  |  |  | 1 |  |  |
| *Keude Bakongan* |  |  |  |  |  |  |  |  |  |  |  | 1 |  |  |  |  |  |  |
| *Kuta Beringin* |  |  |  |  |  |  |  |  |  |  |  | 1 |  |  |  |  |  |  |
| *Labuhan Keude* |  |  |  |  |  |  |  |  | 1 |  |  |  |  |  |  |  |  |  |
| *Lae Bersih* |  |  |  |  |  |  |  |  | 1 |  |  |  |  |  |  |  |  |  |
| *Lawe Sawah* |  |  |  |  |  |  |  |  |  |  |  | 1 |  |  |  |  |  |  |
| *Mane* |  |  |  |  |  |  |  |  | 1 |  |  |  |  |  |  |  |  |  |
| *Namo Buaya* |  |  |  |  |  |  |  |  |  |  |  |  |  |  | 1 |  |  |  |
| *Oboh* |  |  |  |  |  |  |  |  |  |  |  |  |  | 1 |  |  |  |  |
| *Pasar Rundeng* |  |  |  |  |  |  |  |  |  |  |  | 1 |  |  |  |  |  |  |
| *Pasir Putih* |  |  |  |  |  |  |  |  |  |  |  | 1 |  |  |  |  |  |  |
| *Pea Bumbung* |  |  |  |  |  |  |  |  |  |  |  |  |  |  | 1 |  |  |  |
| *Pulo Paya* |  |  |  |  |  |  |  |  |  |  |  |  |  | 1 |  |  |  |  |
| *Rimba Sawang* |  |  |  |  |  |  |  | 9 |  |  |  |  |  |  |  |  |  |  |
| *Sekumur* |  |  |  |  |  |  |  |  |  |  |  |  |  | 1 |  |  |  |  |
| *Sepang* |  |  |  |  |  |  |  |  |  |  |  |  |  |  | 1 |  |  |  |
| *Seuneubok Keuranji* |  |  |  |  |  |  |  |  |  |  | 3 |  |  |  |  |  |  |  |
| *Simpang Kiri* |  |  |  |  |  |  |  | 1 |  |  |  |  | 2 |  |  |  |  |  |
| *Tenggulun* |  |  |  |  |  |  |  | 1 | 2 | 1 | 1 |  |  |  |  |  |  |  |
| *Titi Poben* |  |  |  |  |  |  |  |  |  |  |  |  |  |  | 1 |  |  |  |
| *Ujung Gunung Cut* |  |  |  |  |  |  |  |  |  |  |  |  |  | 2 |  |  |  |  |
| *Ujung Padang* |  |  |  |  |  |  |  |  |  | 4 | 2 | 2 | 6 | 1 |  |  |  |  |
| *Upt Ii Pd Harapan* |  |  |  |  |  |  |  |  |  |  | 1 |  |  |  |  |  |  |  |
| **Cental Kalimantan** | | | | | | | | | | | | | | | | | | |
| *Amin Jaya* |  |  |  |  |  |  |  | 1 |  |  |  |  |  |  |  |  |  |  |
| *Asam Baru* |  |  |  |  |  |  |  |  |  |  |  |  | 2 | 1 |  |  |  |  |
| *Baamang Barat* |  | 48 | 4 |  |  |  |  |  |  |  |  |  |  |  |  |  |  |  |
| *Bagendang Tengah* |  |  |  |  |  |  |  |  |  |  |  |  | 1 |  |  |  |  |  |
| *Baronang II* | 3 |  |  |  |  |  |  |  |  |  |  |  |  |  |  |  |  |  |
| *Baru* |  |  |  |  |  |  |  |  |  |  |  |  |  |  | 1 |  |  |  |
| *Baung* |  | 4 |  |  |  |  |  |  |  |  |  |  |  |  |  |  |  |  |
| *Buntok Kota* |  |  |  |  |  |  |  |  |  |  | 1 |  |  |  |  |  |  |  |
| *Candi* |  |  |  |  |  |  |  |  |  |  |  |  |  | 1 |  |  |  |  |
| *Cempaka Baru* |  |  |  |  |  |  |  |  |  |  | 1 |  |  |  |  |  |  |  |
| *Dadahup* |  | 14 |  |  |  |  |  |  |  |  |  |  |  |  |  |  |  |  |
| *Danau Sembuluh* |  |  |  |  |  | 2 |  |  |  |  |  |  |  |  |  |  |  |  |
| *Gohong* | 1 |  |  |  |  |  |  |  |  |  |  |  |  |  |  |  |  |  |
| *Habaring Hurung* |  |  |  |  |  |  |  | 1 |  |  |  |  |  |  |  |  |  |  |
| *Hampalit* |  |  |  |  |  |  | 1 |  |  |  |  |  |  |  |  |  |  |  |
| *Hiyang Bana* |  |  |  |  |  |  |  |  |  |  |  | 2 |  |  |  |  |  |  |
| *Jahitan* |  | 12 |  |  |  |  |  |  |  |  |  |  |  |  |  |  |  |  |
| *Kalang* |  |  | 1 |  |  |  |  |  |  |  |  |  |  |  |  |  |  |  |
| *Kumai Hulu* |  |  |  |  |  |  |  |  |  | 1 |  |  | 2 |  |  |  |  |  |
| *Lamunti* | 2 |  |  |  |  |  |  |  |  |  |  |  |  |  |  |  |  |  |
| *Marang* |  |  |  |  |  |  |  |  | 1 | 1 |  |  |  |  |  |  |  |  |
| *Mendawai Seberang* |  |  |  |  |  |  |  | 1 |  |  |  |  |  |  |  |  |  |  |
| *Mentawa Baru Hulu* |  |  |  |  |  |  |  | 1 |  |  |  |  | 1 |  |  |  |  |  |
| *Muara Dua* |  |  | 1 |  |  |  |  |  |  |  |  |  |  |  |  |  |  |  |
| *Natai Baru* |  |  |  |  |  |  |  |  |  |  | 1 |  |  |  |  |  |  |  |
| *Natai Raya* |  |  |  |  |  |  |  | 1 |  |  |  |  |  |  |  |  |  |  |
| *Pandu Sanjaya* |  |  |  |  | 1 |  |  |  |  |  |  |  |  |  |  |  |  |  |
| *Parang Batang* |  |  |  |  |  |  |  |  |  |  |  |  |  |  | 1 |  |  |  |
| *Parenggean* | 2 |  |  |  |  |  |  | 1 |  |  |  |  |  |  |  |  |  |  |
| *Patai* |  |  |  |  |  |  |  |  | 3 |  |  |  |  |  |  |  |  |  |
| *Pelantaran* | 2 |  |  |  |  |  |  |  |  |  |  |  |  |  |  |  |  |  |
| *Pembuang Hulu I* |  |  |  |  |  |  |  |  |  |  |  |  | 2 |  |  |  |  |  |
| *Perigi* |  |  |  |  |  |  |  |  |  |  | 6 |  |  |  |  |  |  |  |
| *Petak Bahandang* |  |  |  |  |  |  |  |  |  |  |  | 1 |  |  |  |  |  |  |
| *Petuk Katimpun* |  |  |  |  |  |  |  |  | 1 |  |  |  |  |  |  |  |  |  |
| *Pulua Kaladan /Kaladan Jaya* | 1 |  |  |  |  |  |  |  |  |  |  |  |  |  |  |  |  |  |
| *Pundu* | 8 |  | 1 |  |  |  | 1 | 4 | 6 |  |  |  |  |  |  |  |  |  |
| *Salunuk* |  |  |  |  |  |  |  |  | 1 |  |  |  |  |  |  |  |  |  |
| *Sebabi* | 5 |  |  |  |  |  |  |  |  |  |  |  |  |  |  |  |  |  |
| *Sei Ahas* | 1 |  |  |  |  |  |  |  |  |  |  |  |  |  |  |  |  |  |
| *Selucing* | 3 | 4 |  |  |  |  |  |  |  |  |  |  |  |  |  |  |  |  |
| *Sidorejo* |  |  | 1 |  |  |  |  |  |  |  |  |  |  |  |  |  |  |  |
| *Sumber Makmur* |  |  |  |  |  |  |  |  |  | 1 |  |  |  |  |  |  |  |  |
| *Sungai Kapitan* |  |  |  |  |  |  |  |  |  |  | 4 |  |  |  |  |  |  |  |
| *Sungai Sekonyer* |  |  |  |  |  |  |  |  |  | 1 |  |  |  |  |  |  |  |  |
| *Sungai Tendang* |  |  |  |  |  |  |  |  |  |  |  |  |  |  | 1 |  |  |  |
| *Tamiang Layang* | 1 |  |  |  |  |  |  |  |  |  |  |  |  |  |  |  |  |  |
| *Tangar* |  |  |  |  |  |  |  |  |  |  |  |  | 1 |  |  |  |  |  |
| *Tanjung Hanau* |  |  |  |  |  |  |  |  |  |  | 1 |  |  |  |  |  |  |  |
| *Tanjung Putri* |  |  |  |  | 1 |  |  |  |  |  |  |  |  |  |  |  |  |  |
| *Taringen* |  |  |  |  |  |  |  | 1 |  |  |  |  |  |  |  |  |  |  |
| *Teluk Pulai* |  |  |  |  |  |  |  |  |  |  |  |  |  |  | 1 |  |  |  |
| *Tumbang Kalang* |  |  | 2 | 4 |  |  |  |  |  |  |  |  |  |  |  |  |  |  |
| *Tumbang Mangkutup* | 1 |  |  |  |  |  |  |  |  |  | 76 | 6 | 22 |  | 1 |  |  |  |
| *Tumbang Nusa* |  |  |  |  |  |  |  |  |  |  | 1 |  |  |  |  |  |  |  |
| *Tumbang Tahai* | 1 |  |  |  |  |  |  |  |  |  |  |  |  |  |  |  |  |  |
| **East Kalimantan** | | | | | | | | | | | | | | | | | | |
| *Guntung* |  |  |  |  |  |  |  |  |  |  |  |  |  |  | 1 |  |  |  |
| *Menamang Kanan* |  |  |  |  |  |  |  | 2 |  |  |  |  |  |  |  |  |  |  |
| *Merapun* |  |  |  |  |  |  |  |  | 1 | 3 |  |  |  |  |  |  |  |  |
| *Nehesliah Bing* |  |  |  |  |  |  |  |  |  |  | 1 |  |  |  |  |  |  |  |
| *Paser Utara* |  |  |  |  |  |  |  |  |  |  |  | 1 |  |  |  |  |  |  |
| *Sangatta Selatan* |  |  |  |  |  |  |  |  |  |  |  |  |  | 1 |  |  |  |  |
| *Sangkima* |  |  |  |  |  |  |  | 1 |  |  |  |  |  |  |  |  |  |  |
| *Sangkulirang* |  |  |  |  |  |  |  | 5 | 5 | 5 | 5 |  |  |  |  |  |  |  |
| *Senyiur* |  |  |  |  |  |  |  |  |  |  |  |  |  |  | 1 |  |  |  |
| *Sungai Lesan* |  |  |  |  |  |  |  |  |  |  |  |  | 1 |  |  |  |  |  |
| *Telen* |  |  |  |  |  |  | 4 | 4 | 4 | 4 | 4 |  |  |  |  |  |  |  |
| *Wana Sari* |  |  |  |  |  |  |  | 1 |  |  |  |  |  |  |  |  |  |  |
| *Waru* | 1 |  |  |  |  |  |  |  |  |  |  |  |  |  |  |  |  |  |
| **North Sumatra** | | | | | | | | | | | | | | | | | | |
| *Aek Batang Paya* |  |  |  |  |  |  |  |  |  |  |  |  |  |  | 1 |  |  |  |
| *Besilam* |  |  |  |  |  |  |  |  | 2 |  |  |  |  |  |  |  |  |  |
| *Bukit Lawang* |  |  |  |  |  |  |  | 1 |  |  |  |  |  | 1 |  | 2 |  |  |
| *Bukit Mas* |  |  |  |  |  |  |  |  |  |  | 2 | 1 |  | 1 |  | 2 |  |  |
| *Bukit Selamat* |  |  |  |  |  |  |  |  |  |  |  | 1 | 2 |  |  |  |  |  |
| *Dolok Nauli* |  |  |  |  |  |  |  |  |  |  |  |  |  |  | 1 | 1 |  |  |
| *Halaban* |  |  |  |  |  |  |  |  |  |  | 2 |  |  |  |  |  |  |  |
| *Kuala Musam* |  |  |  |  |  |  |  |  |  |  | 2 |  |  |  |  |  |  |  |
| *Kuta Meriah* |  |  |  |  |  |  |  |  |  |  |  | 1 |  |  |  |  |  |  |
| *Namu Sialang* |  |  |  |  |  |  |  |  |  |  |  |  |  | 1 |  |  |  |  |
| *Padang Bujur* |  |  |  |  |  |  |  |  | 1 |  |  |  |  |  |  |  |  |  |
| *Paya Tusan* |  |  |  |  |  |  |  |  | 1 |  |  |  |  |  |  |  |  |  |
| *Perkebunan Sei Musam* |  |  |  |  |  |  |  |  |  |  | 1 |  |  |  |  |  |  |  |
| *Sampe Raya* |  |  |  |  |  |  |  |  |  |  |  | 2 |  |  |  |  |  |  |
| *Sawit Rejo* |  |  |  |  |  |  |  |  |  | 1 |  |  |  |  |  |  |  |  |
| *Sawit Seberang* |  |  |  |  |  |  |  |  |  |  |  |  | 1 |  |  |  |  |  |
| *Sei Litur Tasik* |  |  |  |  |  |  |  |  | 1 | 1 |  |  |  |  | 1 |  |  |  |
| *Sei Musam* |  |  |  |  |  |  |  |  |  |  |  |  |  | 1 |  |  |  |  |
| *Sei Serdang* |  |  |  |  |  |  |  | 1 | 5 | 1 | 3 | 4 | 3 | 1 |  |  |  |  |
| *Suka Rende* |  |  |  |  |  |  |  |  |  | 1 |  |  |  |  |  |  |  |  |
| *Sulkam* |  |  |  |  |  |  |  |  |  |  |  | 1 |  |  |  |  |  |  |
| *Ujung Bandar* |  |  |  |  |  |  |  |  |  |  |  |  |  |  |  |  | 1 |  |
| **West Kalimantan** | | | | | | | | | | | | | | | | | | |
| *Batu Lintang* | 5 |  |  |  |  |  |  |  |  |  |  |  |  |  |  |  |  |  |
| *Kuala Satong* |  |  |  |  |  |  |  |  |  |  | 2 |  |  |  |  |  |  |  |
| *Kuala Tolak* |  |  |  |  |  |  |  |  |  |  |  |  |  |  | 1 |  |  |  |
| *Laman Satong* |  |  |  |  |  |  |  | 4 |  |  |  |  |  |  |  |  |  |  |
| *Pangkalan Telok* |  |  |  |  |  |  |  |  | 4 |  |  |  |  |  |  |  |  |  |
| *Pematang Gadung* |  |  |  |  |  |  |  |  | 1 |  | 6 |  |  |  |  |  |  |  |
| *Penjalaan* |  |  |  |  |  |  |  |  |  |  | 1 |  |  |  |  |  |  |  |
| *Riam Berasapjaya* |  |  |  |  |  |  |  |  |  |  |  |  | 1 | 1 |  |  |  |  |
| *Simpang Tiga* |  |  |  |  |  |  |  |  |  |  |  | 5 |  |  |  |  |  |  |
| *Sumber Priangan* |  |  |  |  |  |  |  |  |  |  |  | 1 |  |  |  |  |  |  |
| *Sungai Awan Kiri* |  |  |  |  |  |  | 1 |  |  |  | 1 | 1 | 1 |  | 1 |  |  |  |
| *Sungai Mata-Mata* |  |  |  |  |  |  |  |  |  |  |  | 1 |  |  |  |  |  |  |
| *Sungai Pelang* |  |  |  |  |  |  |  |  |  |  |  |  |  |  |  |  | 1 |  |
| *Tanjung Baik Budi* |  |  |  |  |  |  | 2 |  | 1 |  |  |  |  |  |  |  |  |  |
| *Tanjung Pura* |  |  |  |  |  |  |  |  |  |  |  | 1 | 2 |  | 1 |  |  |  |
| *Wajo Hilir* |  |  |  |  |  |  |  | 1 |  |  |  |  |  | 1 |  |  |  |  |
