# Supplementary material for: Outcomes of orangutan wild-to-wild translocations reveal conservation and welfare risks
Source: PLoS One. 2025 Mar 19;20(3):e0317862. doi: 10.1371/journal.pone.0317862 (PMC11970725; doi:10.1371/journal.pone.0317862)
Supplement: S1 Table — Online links were last accessed on December 16, 2024 unless otherwise noted. Searches were limited to the date range of January 1, 2005–December 31, 2022. Some data sources could not be listed due to confidentiality issues. The table does not list webpage links for previously public sources that are no longer available online. We used additional publicly available and unpublished data from individuals and organizations not listed here due to confidentiality concerns. (DOCX) [file pone.0317862.s002.docx]

**S1 Table. List of data sources.** Online links were last accessed on December 16, 2024 unless otherwise noted. Searches were limited to the date range of January 1, 2005 – December 31, 2022. Some data provided cannot be listed due to confidentiality issues. The table does not list webpage links for previously public sources that are no longer available online. We used additional publicly available and unpublished data from individuals and organizations not listed here due to confidentiality concerns.

| **Source type** | **Source name / description** | **Citation or web address** | **Search categories** | **Search terms** |
| --- | --- | --- | --- | --- |
| **Newspaper searches** | ANTARA News | <https://en.antaranews.com/> |  | “orangutan”, “orang-utan” |
|  | Forest Digest | <https://www.forestdigest.com/> |  | “orangutan”, “orang-utan” |
|  | Google advanced search | https://www.google.com/advanced_search | News | “orangutan”, “orang-utan” |
|  | ForestHints | https://foresthints.news/index.html |  |  |
|  | JPNN | https://www.jpnn.com/ |  | “orangutan”, “orang-utan” |
|  | Kompas | <https://www.kompas.com/> |  | “orangutan”, “orang-utan” |
|  | Merdeka | <https://www.merdeka.com/> |  | “orangutan”, “orang-utan” |
|  | Mongabay | <https://news.mongabay.com/> |  | “orangutan”, “orang-utan” |
|  | Mongabay Indonesia | <https://www.mongabay.co.id/> |  | “orangutan”, “orang-utan” |
|  | Okezone | <https://www.okezone.com/> |  | “orangutan”, “orang-utan” |
|  | Prokal | <https://news.prokal.co/> |  | “orangutan”, “orang-utan” |
|  | The Jakarta Post | <https://www.thejakartapost.com/> |  | “orangutan”, “orang-utan” |
|  | Tempo | <https://www.tempo.co/> |  |  |
|  | TribunNews | <https://www.tribunnews.com/> |  | “orangutan”, “orang-utan” |
| **Government sources** | Balai Besar Konservasi Sumber Daya Alam (BBKSDA) North Sumatra | https://bbksdasumut.com/ |  |  |
|  | Balai Konservasi Sumber Daya Alam (BKSDA) East Kalimantan | <http://bksdakaltim.menlhk.go.id/> |  |  |
|  | BKSDA Central Kalimantan | <http://bksda-skw2.blogspot.com/> |  |  |
|  | BKSDA West Kalimantan | <https://ksdakalbar.wordpress.com/> |  |  |
|  | Direktorat Jendral Konservasi Sumber Daya Alam dan Ekosistem (KSDAE) news blog | http://ksdae.menlhk.go.id/pencarian-berita.html |  | “orangutan”, “orang-utan” |
|  | KSDAE annual statistics | http://ksdae.menlhk.go.id/ |  |  |
|  | Kementerian Lingkungan Hidup & Kehutanan (KLHK) | https://www.menlhk.go.id/ |  |  |
|  | Ministry of Forestry | Hutagalung, R. (2018, Feb. 7). KLHK Telah Selamatkan Lebih dari 1.000 Individu Orangutan. *Ministry of Forestry.* No longer available online |  |  |
| **NGO reports, online new blogs and other website source** | Bornean Orangutan Survival Foundation (BOSF) | <https://www.orangutan.or.id/our-latest-stories> |  |  |
|  | BOS Australia – Adopt an orangutan | <https://www.orangutans.com.au/meet-our-family/> |  |  |
|  | BOSF Annual Reports 2006 - 2017 | No longer available online. Previously accessed through BOSF website, www.orangutan.id |  |  |
|  | BOSF Annual Report 2019 | BOSF. (2020). Bornean Orangutan Survival Foundation Annual Report 2019. Bogor, Indonesia. No longer available online. |  |  |
|  | BOS Germany 2008 Annual Report | BOS Deutschland. (2009). Rechenschaftsbericht und Mittelverwendung 2008: Bericht aus den BOS-Projekten, Aktivitäten und Ausblicke 2008 / 2009. Berlin, Germany. |  |  |
|  |  |  |  |  |
|  |  |  |  |  |
|  | BOS Germany 2009 Annual Report | BOS Deutschland. (2010). Rechenschaftsbericht  und Mittelverwendung 2008: Bericht aus den BOS-Projekten,  Aktivitäten und Ausblicke 2008 / 2009. Berlin, Germany. |  |  |
|  |  |  |  |  |
|  |  |  |  |  |
|  | BOS Germany 2010 Annual Report | BOS Deutschland. (2011). Jahresbericht und Mittelverwendung 2010: Bericht aus den BOS-Projekten. Berlin, Germany. |  |  |
|  | BOSF Highlights 2017 | BOSF. (2018). Bornean Orangutan Survival Foundation Highlights 2017. Bogor, Indonesia. No longer available online. |  |  |
|  | BOSF Highlights 2018 | BOSF. (2019). Bornean Orangutan Survival Foundation Highlights 2017. Bogor, Indonesia. No longer available online. |  |  |
|  | BOSF Independent Auditor’s Report 2006-2007 | BOSF. (2008). Independent Auditor’s Report on Financial Statements on the Years Then Ended December 31, 2007 and 2006. HLB Hadori & Rekan, Jakarta, Indonesia. |  |  |
|  | BOSF Independent Auditor’s Report on Financial Statements 2008 and 2009 | BOSF. (2010). Independent Auditor’s Report on Financial Statements on the Years Then Ended December 31, 2008 and 2009. HLB Hadori & Rekan, Jakarta, Indonesia. |  |  |
|  | BOSF Nyaru Menteng Official Release Stories | <https://goingback2dforest.wordpress.com/> |  |  |
|  | BOSF Progress Report | BOS Foundation and RHOI. (2013). Progress Report: Orangutan Post Release Monitoring in Kehje Sewen Forest, East Kalimantan. BOS Foundation and Restorasi Habitat Orangutan Indonesia (RHOI). Bogor, Indonesia. No longer available online. |  |  |
|  | BOSF Progress Report | BOSF. (2015). Progress Report #4: Orangutan Reintroduction and Post-release Monitoring in Bukit Batikap Conservation Forest, Murung Raya August 2013 - December 2014. BOS Foundation - Central Kalimantan Orangutan Reintroduction Program at Nyaru Menteng. Bogor, Indonesia. No longer available online. |  |  |
|  | BOSF Progress report | BOSF. (2016). Progress report: Post-release Monitoring Of orangutans in the Kehje Sewen forest, East Kalimantan 2015. Bogor, Indonesia. No longer available online. |  |  |
|  | BOSF Samboja Lestari & Kehje Sewen Official Release Stories | <https://orangutanforest.wordpress.com/> |  |  |
|  | BOSF Orangutan Reintroduction and Post-release Monitoring | BOSF. (2016). Orangutan Reintroduction and Post-release Monitoring in Bukit Batikap Conservation Forest, Murung Raya Central Kalimantan January 2015 to December 2015. Orangutan Reintroduction Program at Nyaru Menteng. Bogor, Indonesia. |  |  |
|  | BOSF Orangutan Reintroduction and Post-release Monitoring | BOSF. (2018). Orangutan Reintroduction and Post-release Monitoring Update on the Reintroduction Programs in Bukit Batikap Conservation Forest and Bukit Baka Bukit Raya National Park January 2016 to October 2017. No longer available online. |  |  |
|  | Centre for Orangutan Protection (COP) | <http://orangutanprotection.com/> |  |  |
|  | Cikananga Wildlife Center | https://www.cikanangawildlifecenter.com/news/ |  |  |
|  | Conservation Action Network Borneo (CAN) | <https://www.canborneo.id/> |  |  |
|  | Direktorat Jenderal Konservasi Sumber Daya Alam dan Ekosistem (KSDAE) | http://ksdae.menlhk.go.id/pencarian-berita.html (search term “orangutan”) |  |  |
|  | Garda Animalia | <https://gardaanimalia.com/> |  |  |
|  | Global Giving | <https://www.globalgiving.org/> |  | “orangutan” |
|  | International Animal Rescue (IAR) | [https://www.internationalanimalrescue.org/news?currency=USD; https://www.internationalanimalrescue.org/projects/orangutan](https://www.internationalanimalrescue.org/news?currency=USD) |  |  |
|  | Yayasan International Animal Rescue Indonesia (YIARI) | [http://www.internationalanimalrescue.or.id/berita/ No longer available online.](http://www.internationalanimalrescue.or.id/berita/) |  |  |
|  | International Animal Rescue (IAR) Annual Reports and website | [https://www.internationalanimalrescue.org/annual-reports#](https://www.internationalanimalrescue.org/annual-reports# ) |  |  |
|  | IAR Charity Commission Reports | <https://register-of-charities.charitycommission.gov.uk/charity-search/-/charity-details/4029510/accounts-and-annual-returns> |  |  |
|  | Yayasan Jejak Pulang | <https://www.four-paws.org/campaigns-topics/sanctuaries/orangutan-forest-school> |  |  |
|  | Masarang Foundation (Masarang) | https://masarang.eu/about/about-masarang/willie-smits/ |  |  |
|  | Orangutan Foundation International (OFI) Annual Reports | [Orangutan Foundation International. (2010). 2009 Annual Report.; https://orangutan.org/about/annual-report/](https://orangutan.org/about/annual-report/) |  |  |
|  | OFI news blog | <https://orangutan.org/blog/> |  |  |
|  | One Green Planet | <https://www.onegreenplanet.org/channel/news/> |  | “orangutan”, “orang-utan” |
|  | Orangutan Foundation-UK (OF-UK) | <https://www.orangutan.org.uk/blog/> |  |  |
|  | Orangutan Foundation-UK (OF-UK) Charity Commission Reports | <https://register-of-charities.charitycommission.gov.uk/charity-search/-/charity-details/3997163/accounts-and-annual-returns> |  |  |
|  | Orangutan Information Centre (OIC) | <https://orangutancentre.org/news/> |  |  |
|  | Orangutan Kutai Project (Kutai Project) | <https://www.orangutan.com/orangutan-kutai-project-field-update-2016/> |  |  |
|  | Orangutan Republik | <https://www.orangutanrepublik.org/what-we-do/mobile-education-and-conservation-unit-program/> |  |  |
|  | PanEco | https://paneco.ch/en/orangutan-conservation-programme/ |  |  |
|  | People’s Trusts for Endangered Species | <https://ptes.org/wp-content/uploads/2015/01/Indonesia-orangutans-HOC-in-agricultural-landscapes-final-report.pdf>; https://ptes.org/wp-content/uploads/2015/02/Indonesia-orangutans-conflict-resonse-unit-project-update.pdf |  |  |
|  | Pronatura | <https://pronaturafoundation.org/sungai-wain-protection-forest/> |  |  |
|  | Scorpion Illegal Wildlife Trade Monitoring Network | <http://www.scorpionmonitor.org/newslist> |  |  |
|  | Sintang Orangutan Centre (SOC) | <https://soc.or.id/artikel/> |  |  |
|  | Sumatran Orangutan Conservation Programme (SOCP) | <https://www.sumatranorangutan.org/category/media/news/> |  |  |
|  | SOCP, YEL and PanEco annual reports | <https://www.sumatranorangutan.org/category/media/publications/> |  |  |
|  | Sumatra Rainforest Institute | http://sumatranrainforest.org/ Not currently accessible online. https://www.globalgiving.org/projects/orangutan-protection-in-wild-tapanuli/#menu |  |  |
|  | Tasikoko | http://www.tasikoki.org/latest-news/ |  |  |
|  | Wildlife Rescue Centre Jojakarta | https://wrcjogja.org/blog/ |  |  |
|  | Yayasan Ekosistem Lestari (YEL) | <https://www.yel.or.id/environmental-education/> |  |  |
|  | Yayorin | <http://www.yayorin.com/> |  |  |
| **Social media** | Animal Sanctuary Trust Indonesia (ASTI) | https://twitter.com/ASTIndonesia |  |  |
|  | BBKSDA North Sumatra | [https://www.facebook.com/bbksdasumut;](https://www.facebook.com/bbksdasumut) |  |  |
|  | BKSDA Aceh | https://www.instagram.com/bksda_aceh/ |  |  |
|  | BKSDA Central Kalimantan | <https://www.facebook.com/BKSDAKalimantanTengah> |  |  |
|  | BKSDA East Kalimantan | <https://www.facebook.com/bksdakaltim.bksdakaltim> |  |  |
|  | BOSF | <https://www.facebook.com/BOSFoundation/> |  |  |
|  | COP | <https://www.facebook.com/saveordelete/> |  |  |
|  | IAR | [https://www.facebook.com/internationalanimalrescue/.](https://www.facebook.com/internationalanimalrescue/) |  |  |
|  | SOC | <https://www.facebook.com/orangutansintang/> |  |  |
|  | FKL | <https://www.facebook.com/leuserconservationforum> |  |  |
|  | Jakarta Animal Aid Network | https://www.facebook.com/jakartaanimalaidnetwork/ |  |  |
|  | OFI | <https://www.facebook.com/orangutanfoundationinternational/> |  |  |
|  | OF-UK | <https://www.facebook.com/orangutanfndn/> |  |  |
|  | Sumatra Rainforest Institute | <https://www.facebook.com/sumatrarainforestinstitute/about> |  |  |
| **Third party reports** | “On the Trail - Information and analysis bulletin on animal poaching and smuggling” – RobindesBois.org | <https://robindesbois.org/en/category/actualites/> |  |  |
|  | Orangutan Appeal UK Newsletters | <https://www.orangutan-appeal.org.uk/about-us/newsletter-archive> |  |  |
| **FORINA database** | FORINA Orangutan Conflict Database | Previously available at http://forina.or.id/konflik/orangutan.php. No longer available online. |  |  |
