# Supplementary material for: Outcomes of orangutan wild-to-wild translocations reveal conservation and welfare risks
Source: PLoS One. 2025 Mar 19;20(3):e0317862. doi: 10.1371/journal.pone.0317862 (PMC11970725; doi:10.1371/journal.pone.0317862)
Supplement: S2 Table — Data were available for 86 captured and translocated orangutans. Locations were measured as straight line shortest distance between GNSS coordinates, or, where coordinates were not available, from the centroid of the capture village administrative unit (desa) and the centroid of the release area (protected area shape file or desa). (DOCX) [file pone.0317862.s003.docx]

**S2 Table.** **Distance from capture site to release area.** Data were available for 86 orangutans captured and translocated. Locations were measured as straight line shortest distance between GNSS coordinates, or, where coordinates were not available, from the centroid of the capture village administrative unit (*desa*) and the centroid of the release area (protected area shape file or *desa*).

|  | **Distance** | **Count** | **%** | **Median** | **Standard deviation** | **Max** |
| --- | --- | --- | --- | --- | --- | --- |
| **All areas** | 5 km or less | 5 | 5.8% |  |  |  |
|  | 6-10 km | 4 | 4.7% |  |  |  |
|  | 11-20 km | 22 | 25.6% |  |  |  |
|  | 21-35 km | 20 | 23.3% |  |  |  |
|  | 36-50 km | 15 | 17.4% |  |  |  |
|  | 51-75 km | 5 | 5.8% |  |  |  |
|  | 76-100 km | 9 | 10.5% |  |  |  |
|  | more than 100 km | 6 | 7% |  |  |  |
|  |  |  |  | 30.4 | 90.2 | 706.4 |
| **Kalimantan** | 5 km or less | 0 | 0% |  |  |  |
|  | 6-10 km | 0 | 0% |  |  |  |
|  | 11-20 km | 1 | 2.8% |  |  |  |
|  | 21-35 km | 11 | 30.6% |  |  |  |
|  | 36-50 km | 12 | 33.3% |  |  |  |
|  | 51-75 km | 3 | 8.3% |  |  |  |
|  | 76-100 km | 9 | 25% |  |  |  |
|  | >100 km | 0 | 0% |  |  |  |
|  |  |  |  | 47.5 | 25.5 | 98.3 |
| **Sumatra** | 5 km or less | 5 | 10% |  |  |  |
|  | 6-10 km | 4 | 8% |  |  |  |
|  | 11-20 km | 21 | 42% |  |  |  |
|  | 21-35 km | 9 | 18% |  |  |  |
|  | 36-50 km | 3 | 6% |  |  |  |
|  | 51-75 km | 2 | 4% |  |  |  |
|  | 76-100 km | 0 | 0% |  |  |  |
|  | more than 100 km | 6 | 12% |  |  |  |
|  |  |  |  | 13.6 | 116.2 | 706.4 |
