# Supplementary material for: Outcomes of orangutan wild-to-wild translocations reveal conservation and welfare risks
Source: PLoS One. 2025 Mar 19;20(3):e0317862. doi: 10.1371/journal.pone.0317862 (PMC11970725; doi:10.1371/journal.pone.0317862)
Supplement: S3 Table — Sources: Records of captures for translocation (this study), published literature, and communications with orangutan researchers and translocation practitioners. (DOCX) [file pone.0317862.s004.docx]

**S2 Table. Habitat types and crops where orangutans were encountered in Kalimantan and Sumatra, 2005-2022.** Sources: Records of captures for translocation (this study), published literature, and communications with orangutan researchers and translocation practitioners.

| **Habitat description** |
| --- |
| Mixed primary and degraded forest |
| Mangrove forest |
| Degraded forest |
| Forest patches isolated from other forest areas |
| Plantations with small fragments of forest, or even just a few tall trees |
| Plantations without forests but near forests and forest fragments |
| Industrial tree plantations |
| Cropland or farms at protected forest edges |
| Cropland or farms with fragmented or isolated forest patches |
| Near villages with fragmented forest patches |
| Agroforest 5 km or more from natural forest patches |
| Boundary areas between forest and plantations, farms, infrastructure, villages, suburban housing, or other human development |
| **Crop types and settings (industrial or small holder plantations/farms)** |
| Banana fruits (*Musa* sp.) - smallholder |
| Coconut palms (Cocos nucifera) - smallholder |
| Durian fruits (*Durio* sp.) - smallholder |
| Jackfruit (*Artocarpus heterophyllus*) - smallholder |
| Jernang fruit (Daemonorops sp.) - smallholder |
| Mangosteen fruit (*Garcinia mangostana*)- smallholder |
| Petai fruit (*Parkia speciosa*) - smallholder |
| Rubber trees (*Hevea brasiliensis*) - smallholder |
| Rambutan fruit (*Nephelium lappaceum*) - smallholder |
| Oil palm (*Elaeis guineensis*) plantations - smallholder |
| Salak fruit (*Salacca zalacca*) - smallholder |
| Rubber tree (*Hevea brasiliensis*) plantations - industrial |
| Eucalyptus tree (*Eucalyptus* sp.) plantations - industrial |
| Acacia tree (*Acacia* sp.) plantations - industrial |
| Albizia tree (*Albizia* sp.) plantations - industrial |
| Oil palm (*Elaeis guineensis*) plantations - industrial |
