# Supplementary material for: Outcomes of orangutan wild-to-wild translocations reveal conservation and welfare risks
Source: PLoS One. 2025 Mar 19;20(3):e0317862. doi: 10.1371/journal.pone.0317862 (PMC11970725; doi:10.1371/journal.pone.0317862)
Supplement: S4 Table — Population figures are based on most recent published estimates in the listed sources. (DOCX) [file pone.0317862.s005.docx]

**S4 Table. Orangutan population estimates and mean annual rate of decline.** Population figures are based on most recent published estimates in the listed sources.

| **Island** | **Area/species** | **Time period** | **Initial population size** | **Latest population size** | **Annual decline (%)**^1^ | **Mean current population size** | **Mean annual decline** | **Mean annual % decline** | **Source** |
| --- | --- | --- | --- | --- | --- | --- | --- | --- | --- |
| Borneo^2^ | Borneo (*Pongo pygmaeus*) | 2000-2015 | 100,000 | 75,000 | 1,667 (2.22%) | 83,575 | 2,788 (SD +1817) | 3.14% | [Santika, Ancrenaz [1]](#_ENREF_1); Supp. Materials Fig. 3 |
|  |  | 2007-2019 | 163,656 | 105,033 | 4,885 (4.65%) |  |  |  | [Voigt, Wich [2]](#_ENREF_2); 2017-2019 extrapolation from [Sherman, Voigt [3]](#_ENREF_3) |
|  |  | 1997-2014 | 101,489 | 70,691 | 1,812 (2.56%) |  |  |  | [Utami-Atmoko, Traylor-Holzer [4]](#_ENREF_4); habitat extrapolation |
|  | Central Kalimantan | 1997-2014 | 49,467 | 34,673 | 870 (2.51%) | 47,512 | 1,536 (SD +942) | 2.54% | [Voigt, Wich [2]](#_ENREF_2); 2017-2019 extrapolation from [Sherman, Voigt [3]](#_ENREF_3) |
|  |  | 2007-2019 | 86,778 | 60,351 | 2,202 (2.58%) |  |  |  | [Utami-Atmoko, Traylor-Holzer [4]](#_ENREF_4); GIS model |
|  | East Kalimantan | 1997-2014 | 30368 | 22847 | 442 (1.94%) | 14,091 | 303 (SD +197) | 2.05% | Voigt et al. 2018; 2017-2019 extrapolation from Sherman et al. 2022 |
|  |  | 2007-2019 | 7294 | 5335 | 163 (2.16%) |  |  |  | [Utami-Atmoko, Traylor-Holzer [4]](#_ENREF_4); GIS model |
|  | West Kalimantan | 1997-2014 | 46,510 | 32,494 | 824 (2.54%) | 25,079 | 813 (SD +16) | 2.87% | [Voigt, Wich [2]](#_ENREF_2); 2017-2019 extrapolation from [Sherman, Voigt [3]](#_ENREF_3) |
|  |  | 2007-2019 | 27,291 | 17,663 | 802 (3.21%) |  |  |  | Utami-Atmoko et al. 2017; GIS model |
| Sumatra^3^ | Sumatra (*Pongo abelii* and *Pongo tapanuliensis*) | 2010-2014 | 14,613 | 10,637 | 398 (3.74%) | 11,861 | 242 (SD +147) | 2.17% | [Wich, Singleton [5]](#_ENREF_5); multi-model estimate scenario 1; 1 km barrier |
|  |  | 2010-2014 | 14,613 | 13,085 | 153 (1.17%) |  |  |  | [Wich, Singleton [5]](#_ENREF_5); multi-model estimate scenario 2; 1 km barrier |
|  |  | 2010-2014 | 13,938 | 10,637 | 330 (3.10%) |  |  |  | [Wich, Singleton [5]](#_ENREF_5); design estimate scenario 1; 5 km barrier |
|  |  | 2010-2014 | 13,938 | 13,085 | 85 (0.65%) |  |  |  | [Wich, Singleton [5]](#_ENREF_5); design estimate scenario 2; 5 km barrier |

1. Estimated annual declines = initial population size – latest population size/years.
2. Borneo figures based on population estimates from: 1) [Santika, Ancrenaz [1]](#_ENREF_1), Supplementary materials Figure 3, [Voigt, Wich [2]](#_ENREF_2) estimates per province used in [Sherman, Voigt [3]](#_ENREF_3), and the GIS model estimates by province in [Utami-Atmoko, Traylor-Holzer [4]](#_ENREF_4).
3. Sumatra estimates are based on the outcome of deforestation scenarios in [Wich, Singleton [5]](#_ENREF_5), using both the 1 km and 5 km dispersal barriers using an initial population per the mean from the multi-model and design estimates, respectively. 2020 populations are based on the estimated numbers for island-wide populations in [Wich, Singleton [5]](#_ENREF_5), Table 3 numbers for “Total abundance”.

Sources: 1. Santika T, Ancrenaz M, Wilson KA, Spehar S, Abram N, Banes GL, et al. First integrative trend analysis for a great ape species in Borneo. Sci Rep. 2017;7(1):4839. doi: 10.1038/s41598-017-04435-9.

2. Voigt M, Wich SA, Ancrenaz M, Meijaard E, Abram N, Banes GL, et al. Global Demand for Natural Resources Eliminated More Than 100,000 Bornean Orangutans. Curr Biol. 2018;28(5):761-9.e5. doi: 10.1016/j.cub.2018.01.053.

3. Sherman J, Voigt M, Ancrenaz M, Wich SA, Qomariah IN, Lyman E, et al. Orangutan killing and trade in Indonesia: Wildlife crime, enforcement, and deterrence patterns. Biol Conserv. 2022;276:109744. doi: 10.1016/j.biocon.2022.109744.

4. Utami-Atmoko S, Traylor-Holzer K, Rifqi MA, Siregar PG, Achmad B, Priadjati A, et al. Orangutan Population and Habitat Viability Assessment: Final Report. Apple Valley, MN.: 2017.

5. Wich SA, Singleton I, Nowak MG, Utami Atmoko SS, Nisam G, Arif SM, et al. Land-cover changes predict steep declines for the Sumatran orangutan (*Pongo abelii*). Science Advances. 2016;2(3):e1500789.
