# Supplementary material for: Outcomes of orangutan wild-to-wild translocations reveal conservation and welfare risks
Source: PLoS One. 2025 Mar 19;20(3):e0317862. doi: 10.1371/journal.pone.0317862 (PMC11970725; doi:10.1371/journal.pone.0317862)
Supplement: S5 Table — Data on desa locations were available for 528 of the 988 reported captures during the study period. F = female, M = male. Estimated age classes are: infant (I) 0–6 years, juvenile/adolescent (J) 7–12 years, adult (A) 15 or more years, and unknown (U). Unknown sex or age means these data were not available in the records we reviewed. Age and sex data may have been collected but not made available in the records we were able to access. (DOCX) [file pone.0317862.s006.docx]

**S5 Table.** **Village administrative units (*desa*) where 7 or more orangutans were reported as captured for wild-to-wild translocation between 2005 and 2022 (Kalimantan) and 2012 to 2022 (Sumatra).** Data on *desa* locations was available for 528 of the 988 reported captures during the study period. F= female, M = male. Estimated age classes are: infant (I) 0-6 years, juvenile/adolescent (J) 7-12 years, adult (A) 15 or more years, and unknown (U). Unknown sex or age means these data were not available in the records we reviewed. Age and sex data may have been collected but not made available in the records we were able to access.

| **Village (Desa) name**^1^ | **Province** | **Island** | **# of Orangutans captured 2005-2022**^2^ | **Sex and estimated age class of captured orangutans** | **Year(s) of capture** |
| --- | --- | --- | --- | --- | --- |
| Ujung Padang | Aceh | Sumatra | 15 | F: A = 6, J = 1  M: A = 4, I = 4 | 2014-2018 |
| Rimba Sawang | Aceh | Sumatra | 9 | F: A = 7, I = 1  M: I = 2 | 2012 |
| Sei Serdang | North Sumatra | Sumatra | 18 | F: A = 9, J = 2  M: A = 2, J = 2, I = 3 | 2012-2018 |
| Tumbang Mangkutup | Central Kalimantan | Borneo | 106 | F: A = 20, I = 4  M: A = 11, J = 1, I = 10  7 Adults unknown sex  4 Infants unknown sex  49 unknown age class + sex | 2005(1), 2015-2019 |
| Baambang Barat | Central Kalimantan | Borneo | 52 | F: A = 14, J = 9, I = 7, U = 3  M: A = 10, J = 3, I = 4, U =2 | 2006-2007 |
| Dadahup | Central Kalimantan | Borneo | 14 | F: A = 2, J = 4, I = 3  M: A = 2, J = 2, I = 1 | 2006 |
| Jahitan | Central Kalimantan | Borneo | 12 | F: A= 2, J= 5, I= 2  M: A = 1, J= 1, I = 1 | 2006 |
| Pundu | Central Kalimantan | Borneo | 20 | F: A = 2, J = 4, I = 5  M: A = 4, J = 3, I = 1, U = 1 | 2005-2013 |
| Sangkulirang | East Kalimantan | Borneo | 20 | Age class + sex unknown | 2012-2015 |
| Telen | East Kalimantan | Borneo | 20 | Age class + sex unknown | 2011-2015 |
| Pematang Gadung | West Kalimantan | Borneo | 7 | F: A =1  M: A= 2  1 Infant unknown sex  3 unknown age class + sex | 2013-2015 |

1. D*esa* are administrative units designated by the Indonesian government. Each *desa* has unique boundaries, geographic extent, biophysical characteristics, and human impacts. Orangutan habitats and populations thus vary among *desa*.
2. Numbers are an underestimate of orangutans captured for translocation as they are based on available records collected for this study. Not all captures for translocation are reported, and not all records were available to authors. Figures for Sumatra represent data from 2012-2022, as no records of captures were available for prior years.
