# Supplementary figures and images for: Outcomes of orangutan wild-to-wild translocations reveal conservation and welfare risks
Source: PLoS One. 2025 Mar 19;20(3):e0317862. doi: 10.1371/journal.pone.0317862 (PMC11970725; doi:10.1371/journal.pone.0317862)

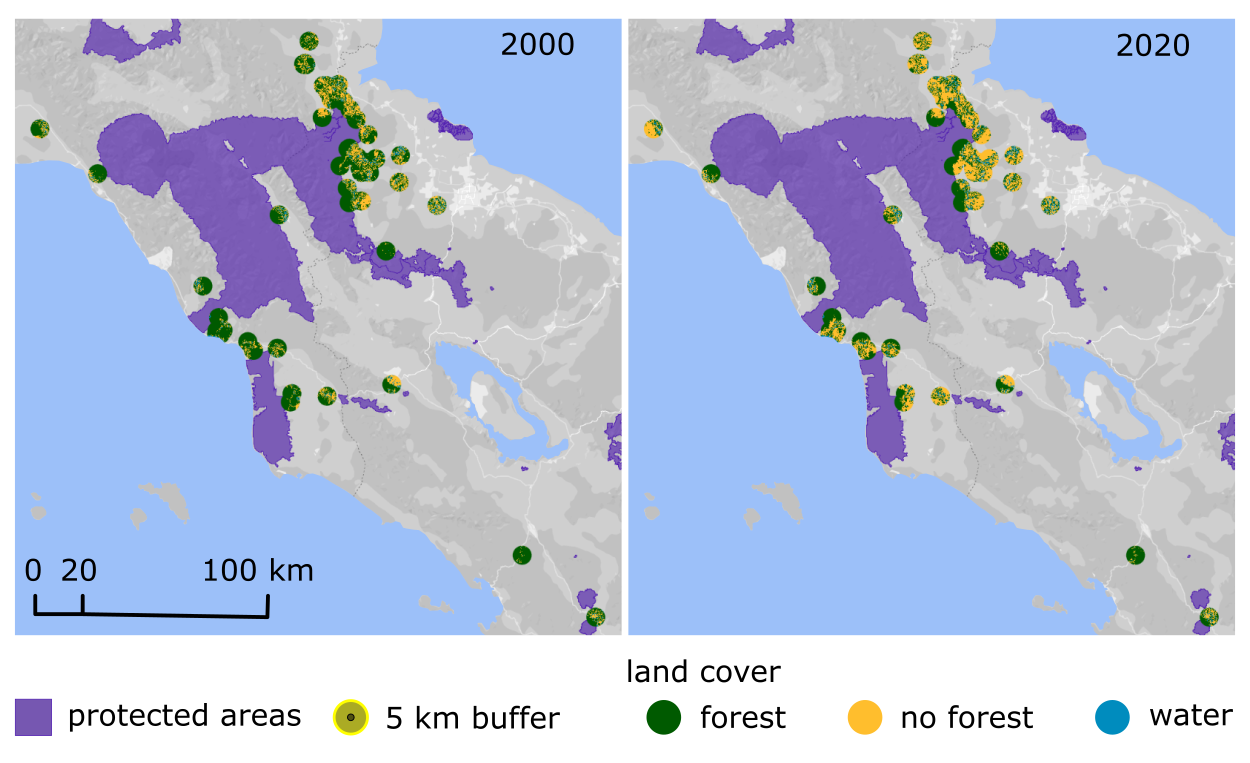

Supplement: S1 Fig — Depicted areas cover a 5-km buffer around GNSS coordinates of sites where 99 orangutans were captured for translocation between 2000 and 2022. Intact tropical moist forest, degraded tropical moist forest, and regrowth from Vancutsem et al. [57] were combined into a single forest class (green) to visualize forested areas available to orangutans. Several captures were reported within the same areas and thus have overlapping buffers. Protected areas (purple): UNEP-WCMC and IUCN [60]. Forest map: EC JRC. Basemap: Google Earth Engine. (TIF) [file pone.0317862.s007.tif]

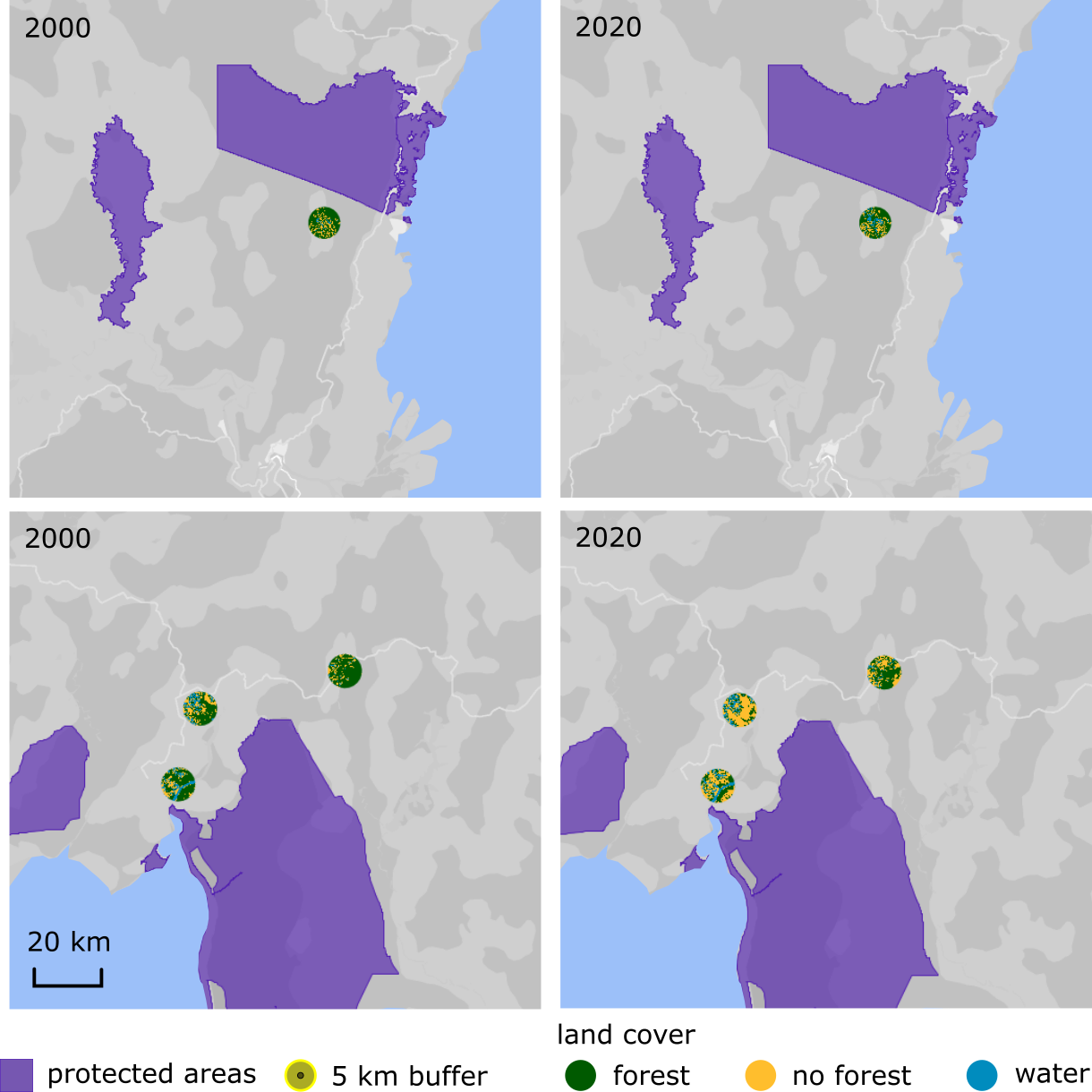

Supplement: S2 Fig — Land cover changes are shown for a 5-km buffer around GNSS coordinates of four sites where five orangutans were captured for translocation between 2000 and 2022. Top panels show one capture site in East Kalimantan. Bottom panels show three capture sites in Central Kalimantan. Intact tropical moist forest, degraded tropical moist forest, and regrowth from Vancutsem et al. [57] were combined into a single forest class (green) to visualize forested areas available to orangutans. Protected areas (purple): UNEP-WCMC and IUCN [60]. Forest map: EC JRC. Basemap: Google Earth Engine. (TIF) [file pone.0317862.s008.tif]

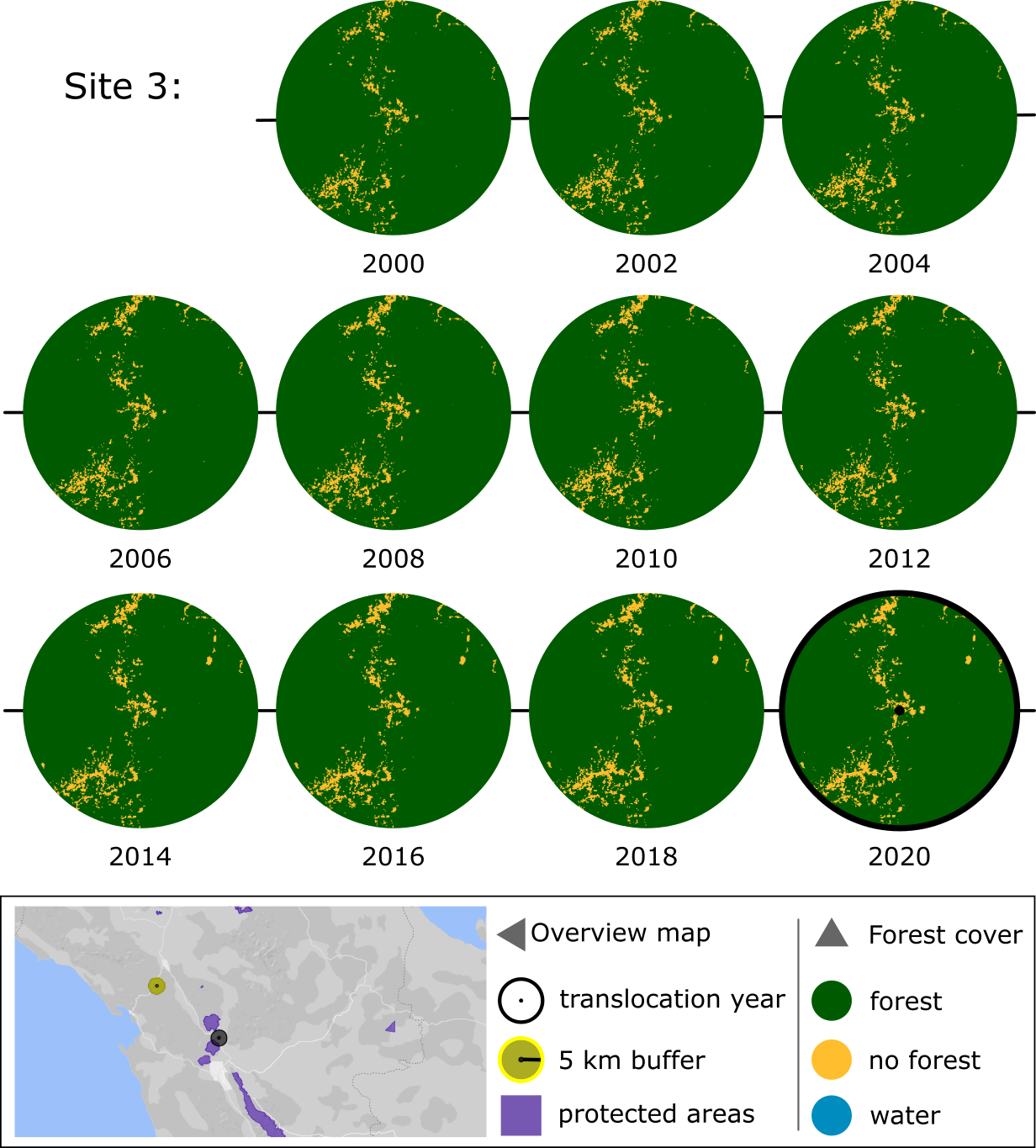

Supplement: S3 Fig — Recapture of a healthy Tapanuli adult male originally captured and translocated in 2019. He had returned to the original capture area and was seen alongside a road. Year of capture (2020) is indicated with a thick black bordered circle. Intact tropical moist forest, degraded tropical moist forest, and regrowth are combined into a single forest class (green). The map inset at the bottom is the location of the specific capture site and buffer (yellow) along with remaining locations where 1 other orangutan was captured for translocation (black). Basemap: Google Earth Engine. Forest map: EC JRC. Protected areas (purple): UNEP-WCMC and IUCN [60]. (TIF) [file pone.0317862.s009.tif]

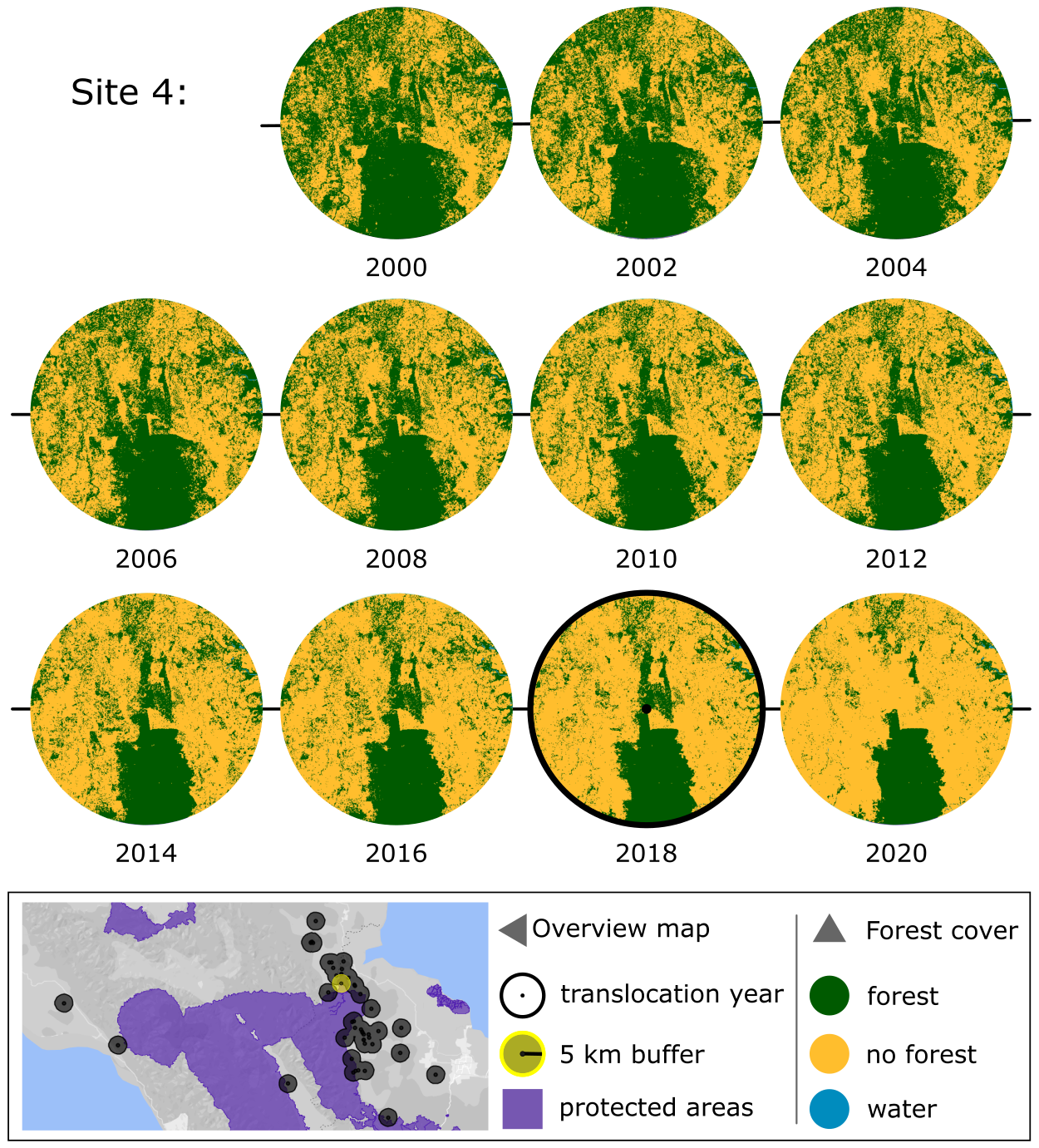

Supplement: S4 Fig — An injured Sumatran adult male reported as seasonally foraging in durian (Durio sp.) and langsat (Lansium parasiticum) gardens at the edge of protected forest. Year of capture (2018) is indicated with thick black bordered circle. Intact tropical moist forest, degraded tropical moist forest, and regrowth are combined into a single forest class (green). The map inset at the bottom is the location of the specific capture site and buffer (yellow) along with remaining locations where 98 other orangutans were captured for translocation (black). Basemap: Google Earth Engine. Forest map: EC JRC. Protected areas (purple): UNEP-WCMC and IUCN [60]. (TIF) [file pone.0317862.s010.tif]

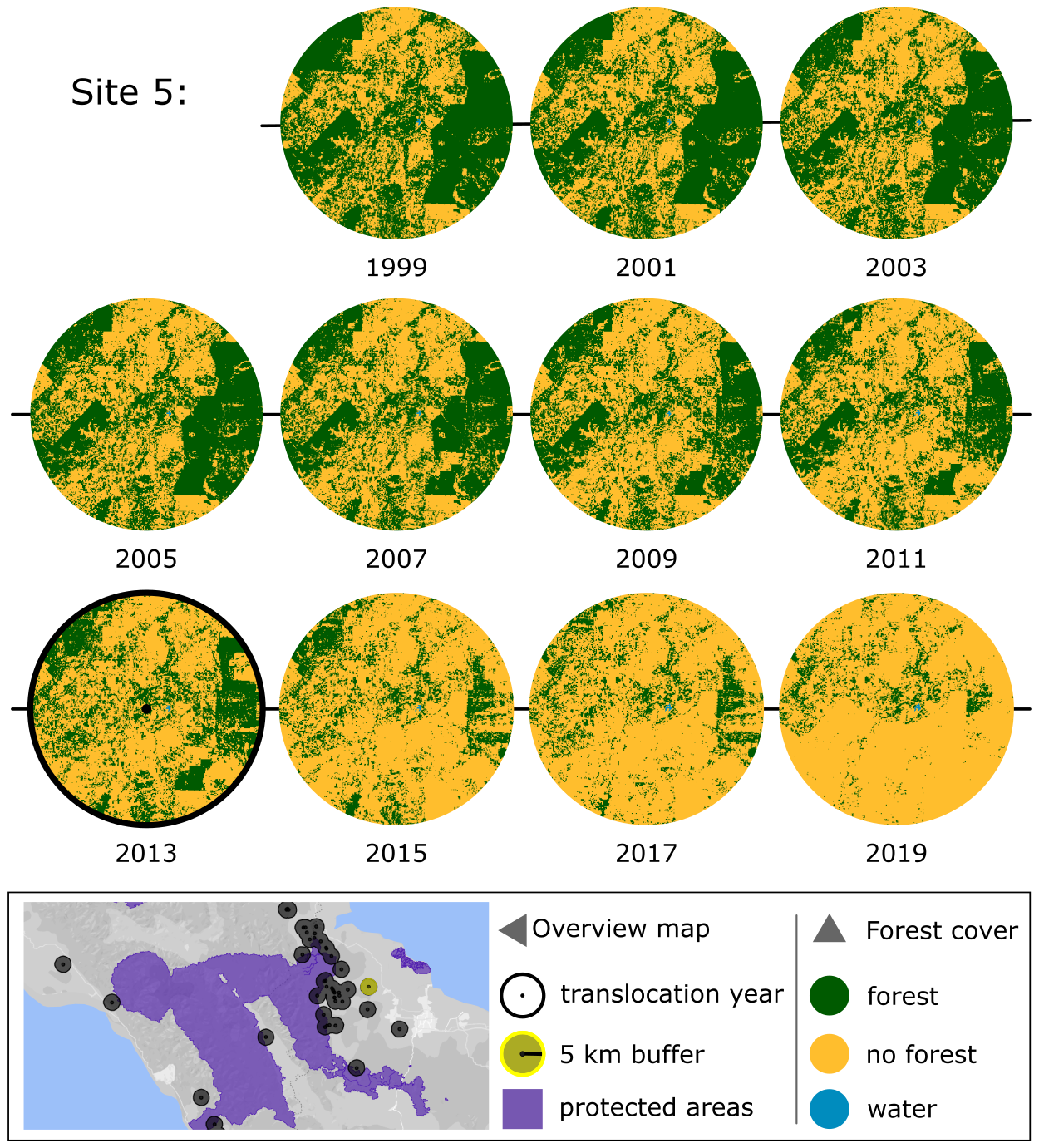

Supplement: S5 Fig — A Sumatran orangutan population reported as isolated in area considered at risk for deforestation; two healthy adult females were translocated from this site. Year of capture (2013) is indicated with thick black bordered circle. Intact tropical moist forest, degraded tropical moist forest, and regrowth are combined into a single forest class (green). The map inset at the bottom is the location of the specific capture site and buffer (yellow) along with remaining locations where 97 other orangutans were captured for translocation (black). Basemap: Google Earth Engine. Forest map: EC JRC. Protected areas (purple): UNEP-WCMC and IUCN [60]. (TIF) [file pone.0317862.s011.tif]

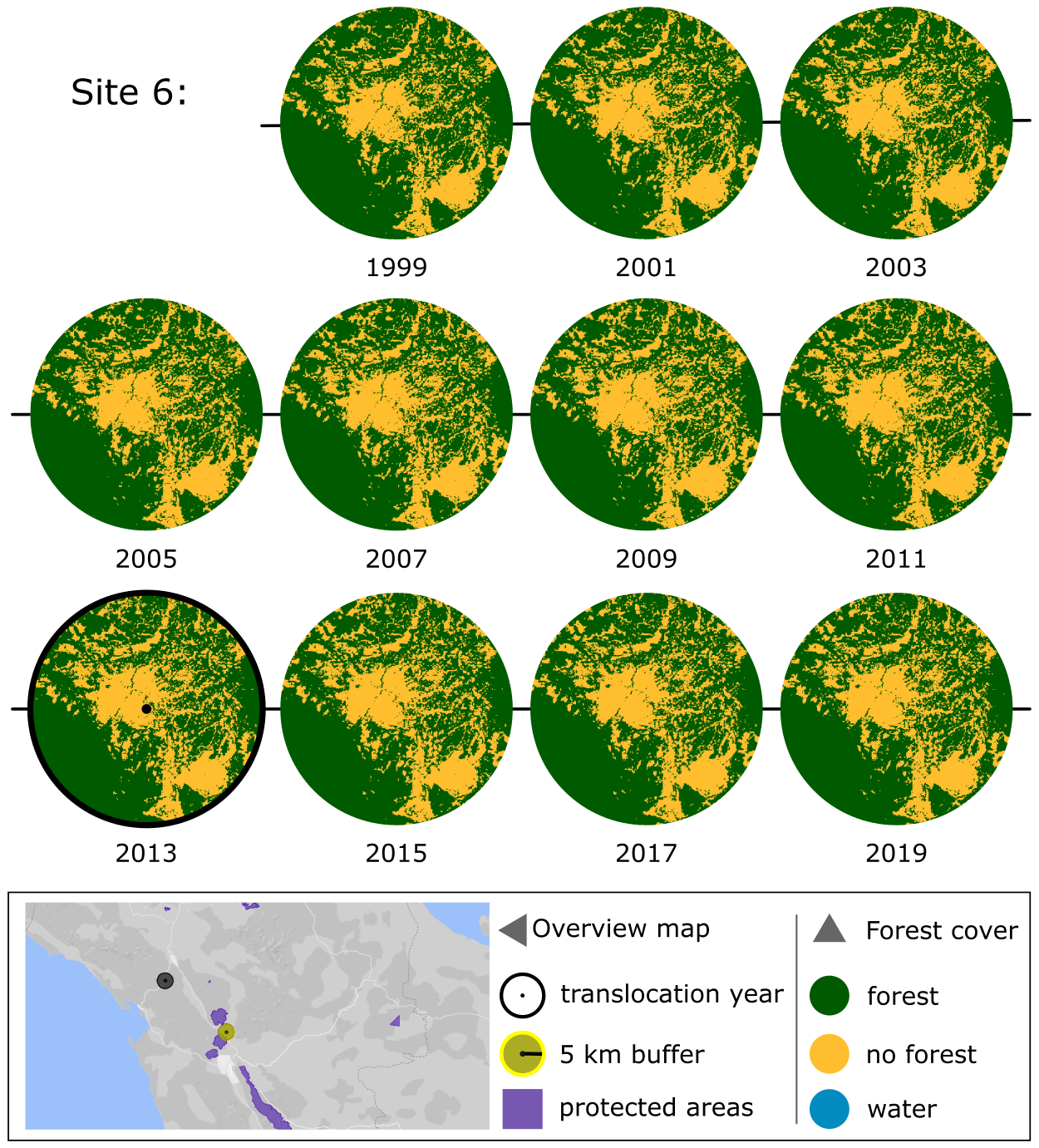

Supplement: S6 Fig — An injured Tapanuli adult male in a salak fruit (Salacca zalacca) plantation. Year of capture (2013) is indicated with thick black bordered circle. Intact tropical moist forest, degraded tropical moist forest, and regrowth are combined into a single forest class (green). The map inset at the bottom is the location of the specific capture site and buffer (yellow) along with remaining locations where 1 other orangutan was captured for translocation (black). Basemap: Google Earth Engine. Forest map: EC JRC. Protected areas (purple): UNEP-WCMC and IUCN [60]. (TIF) [file pone.0317862.s012.tif]

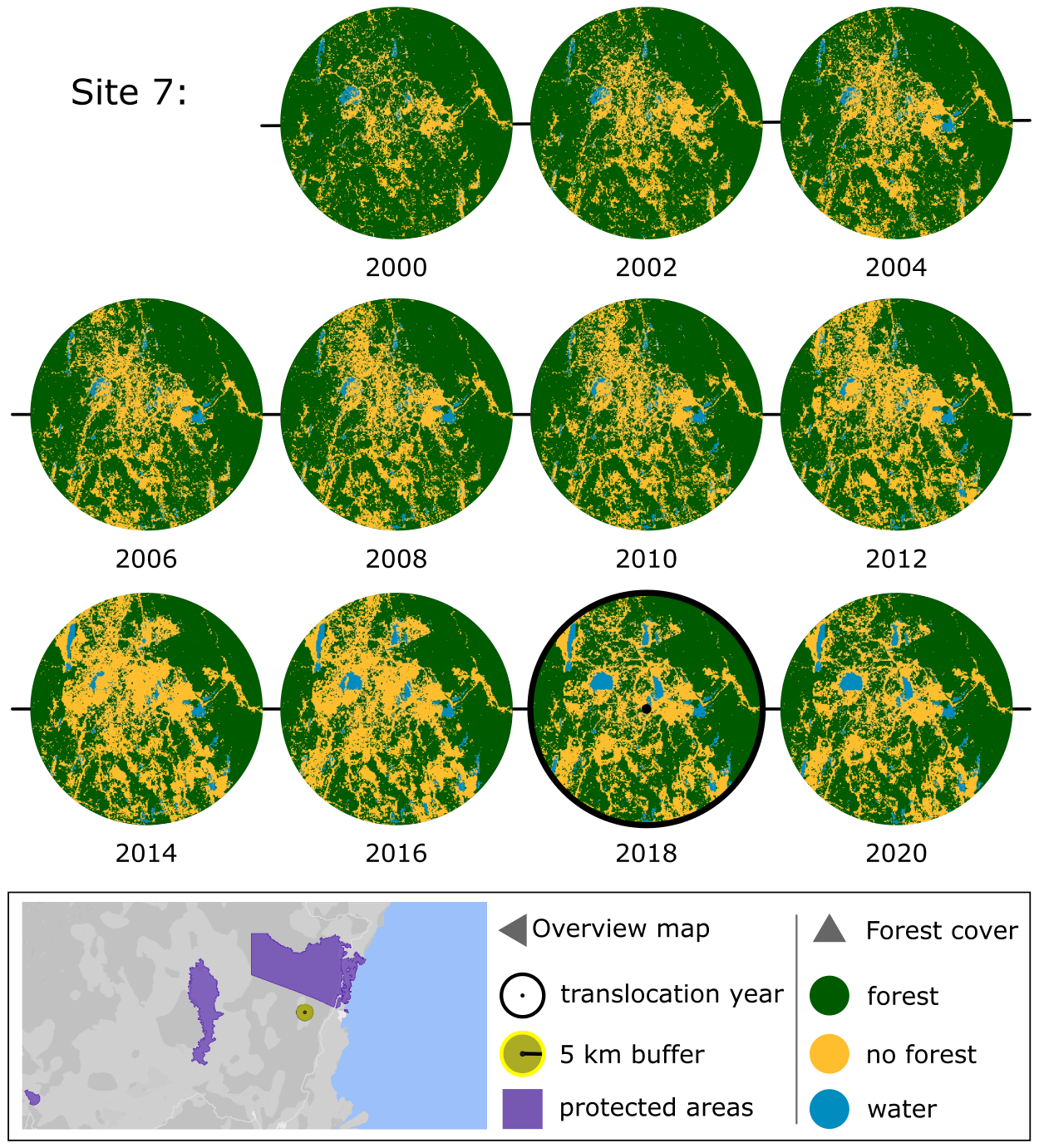

Supplement: S7 Fig — A gravely injured juvenile male Bornean orangutan, previously thought to have been foraging in a local pineapple garden within Kutai National Park. Year of capture (2018) is indicated with thick black bordered circle. Intact tropical moist forest, degraded tropical moist forest, and regrowth are combined into a single forest class (green). The map inset at the bottom is the location of the specific capture site and buffer (yellow). Basemap: Google Earth Engine. Forest map: EC JRC. Protected areas (purple): UNEP-WCMC and IUCN [60]. (TIF) [file pone.0317862.s013.tif]

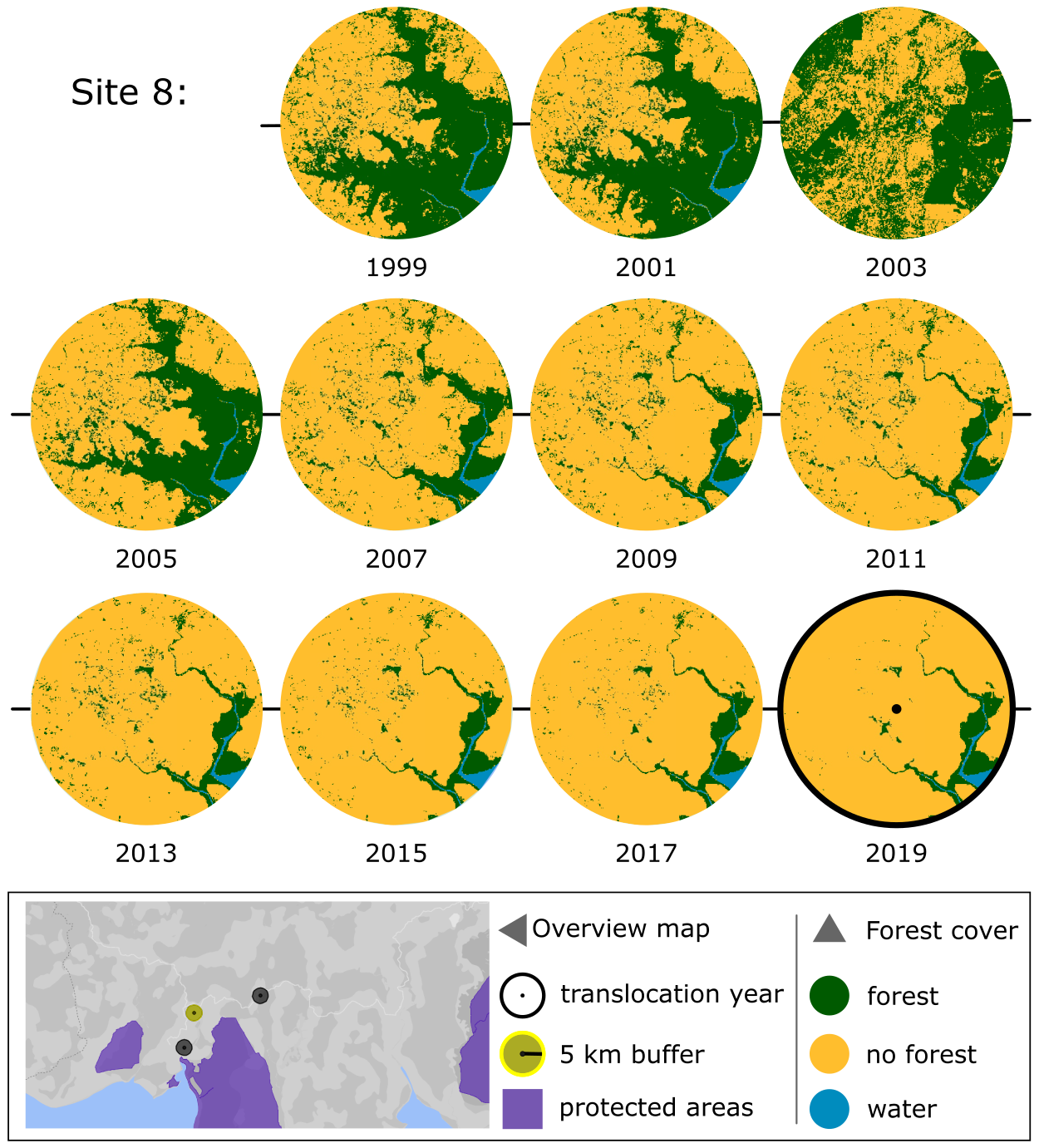

Supplement: S8 Fig — Healthy Bornean adult male reported to be staying in and “disturbing” a local durian garden. Year of capture (2019) is indicated with thick black bordered circle. Intact tropical moist forest, degraded tropical moist forest, and regrowth are combined into a single forest class (green). The map inset at the bottom is the location of the specific capture site and buffer (yellow) along with remaining locations where three other orangutans were captured for translocation (black). Basemap: Google Earth Engine. Forest map: EC JRC. Protected areas (purple): UNEP-WCMC and IUCN [60]. (TIF) [file pone.0317862.s014.tif]

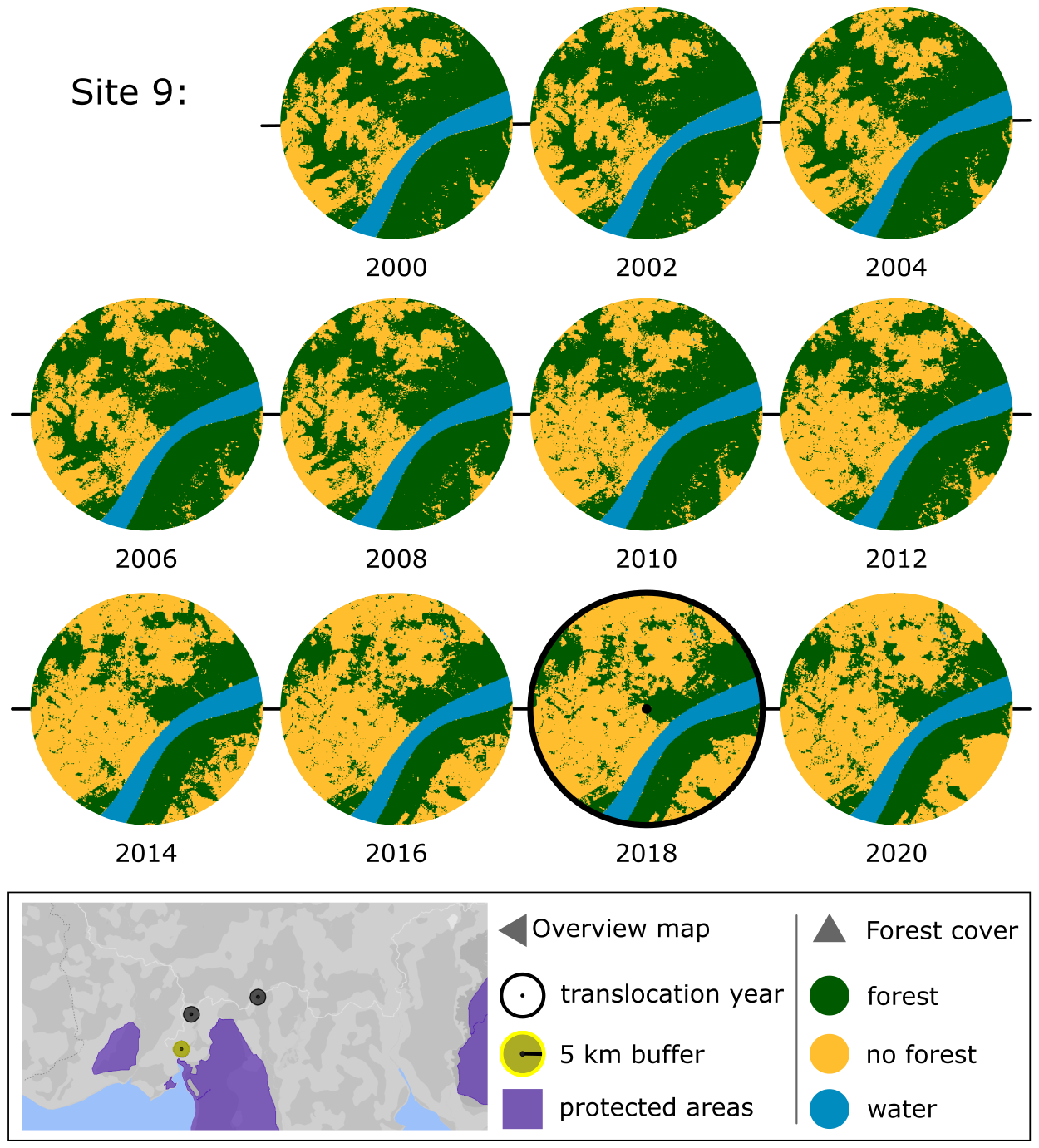

Supplement: S9 Fig — Healthy Bornean adult male seen in APL (non-forest) land near a power plant and uninsulated electrical wires. Year of capture (2018) is indicated with thick black bordered circle. Intact tropical moist forest, degraded tropical moist forest, and regrowth are combined into a single forest class (green). The map inset at the bottom is the location of the specific capture site and buffer (yellow) along with remaining locations where three other orangutans were captured for translocation (black). Basemap: Google Earth Engine. Forest map: EC JRC. Protected areas (purple): UNEP-WCMC and IUCN [60]. (TIF) [file pone.0317862.s015.tif]
